# Biophysical network modeling of the dLGN circuit: Effects of cortical feedback on spatial response properties of relay cells

**DOI:** 10.1371/journal.pcbi.1005930

**Published:** 2018-01-29

**Authors:** Pablo Martínez-Cañada, Milad Hobbi Mobarhan, Geir Halnes, Marianne Fyhn, Christian Morillas, Francisco Pelayo, Gaute T. Einevoll

**Affiliations:** 1 Department of Computer Architecture and Technology, University of Granada, Granada, Spain; 2 Centro de Investigación en Tecnologías de la Información y de las Comunicaciones (CITIC), University of Granada, Granada, Spain; 3 Center for Integrative Neuroplasticity (CINPLA), University of Oslo, Oslo, Norway; 4 Department of Biosciences, University of Oslo, Oslo, Norway; 5 Faculty of Science and Technology, Norwegian University of Life Sciences, Ås, Norway; 6 Department of Physics, University of Oslo, Oslo, Norway; University College London, UNITED STATES

## Abstract

Despite half-a-century of research since the seminal work of Hubel and Wiesel, the role of the dorsal lateral geniculate nucleus (dLGN) in shaping the visual signals is not properly understood. Placed on route from retina to primary visual cortex in the early visual pathway, a striking feature of the dLGN circuit is that both the relay cells (RCs) and interneurons (INs) not only receive feedforward input from retinal ganglion cells, but also a prominent feedback from cells in layer 6 of visual cortex. This feedback has been proposed to affect synchronicity and other temporal properties of the RC firing. It has also been seen to affect spatial properties such as the center-surround antagonism of thalamic receptive fields, i.e., the suppression of the response to very large stimuli compared to smaller, more optimal stimuli. Here we explore the spatial effects of cortical feedback on the RC response by means of a a comprehensive network model with biophysically detailed, single-compartment and multicompartment neuron models of RCs, INs and a population of orientation-selective layer 6 simple cells, consisting of pyramidal cells (PY). We have considered two different arrangements of synaptic feedback from the ON and OFF zones in the visual cortex to the dLGN: phase-reversed (‘push-pull’) and phase-matched (‘push-push’), as well as different spatial extents of the corticothalamic projection pattern. Our simulation results support that a phase-reversed arrangement provides a more effective way for cortical feedback to provide the increased center-surround antagonism seen in experiments both for flashing spots and, even more prominently, for patch gratings. This implies that ON-center RCs receive direct excitation from OFF-dominated cortical cells and indirect inhibitory feedback from ON-dominated cortical cells. The increased center-surround antagonism in the model is accompanied by spatial focusing, i.e., the maximum RC response occurs for smaller stimuli when feedback is present.

## Author summary

The functional role of the dorsal lateral geniculate nucleus (dLGN), placed on route from retina to primary visual cortex in the early visual pathway, is still poorly understood. A striking feature of the dLGN circuit is that dLGN cells not only receive feedforward input from the retina, but also a prominent feedback from cells in the visual cortex. It has been seen in experiments that cortical feedback modifies the spatial properties of dLGN cells in response to visual stimuli. In particular, it has been shown to increase the center-surround antagonism for flashing-spot and patch-grating visual stimuli, i.e., the suppression of responses to very large stimuli compared to smaller stimuli. Here we investigate the putative mechanisms behind this feature by means of a comprehensive network model of biophysically detailed neuron models for RCs and INs in the dLGN and orientation-selective cortical cells providing the feedback. Our results support that the experimentally observed feedback effects may be due to a phase-reversed (‘push-pull’) arrangement of the cortical feedback where ON-symmetry RCs receive (indirect) inhibitory feedback from ON-dominated cortical cell and excitation from OFF-dominated cortical cells, and vice versa for OFF-symmetry RCs.

## Introduction

Visual signals from the retina pass through the dorsal geniculate nucleus (dLGN), the visual part of thalamus, on the way to the visual cortex. However, this is not simply a one-way flow of information: cortical cells feed back to both relay cells (RCs) and interneurons (INs) in the dLGN and thus shape the transfer of visual information in the circuit [1–6]. Although there is no broad consensus about the effects of cortical feedback on sensory processing, there are many experimental studies that provide insight into its potential roles [7–20]. For example, cortical feedback has been observed to switch the response mode of RCs between tonic and burst modes [21, 22] and to synchronize the firing patterns of groups of dLGN cells [17]. Further, the studies have reported both enhanced and reduced responses of dLGN neurons from cortical feedback, and the functional role of cortical feedback is still debated [3, 23, 24].

One line of inquiry has addressed the question of how cortical feedback modulates the receptive-field properties of RCs. Cortical feedback was early shown to affect the length tuning of RC responses [12], and a series of studies from Sillito and co-workers have investigated how cortical feedback influences the RC responses to flashing spots and patch gratings, i.e., circular patches of drifting gratings [4, 13, 15, 16, 18, 19]. Retinal ganglion cells (GCs) provide the feedforward input to the dLGN circuit, and the receptive fields of both GCs and RCs have a roughly circular shape where an excitatory center is surrounded by an inhibitory surround [25–27]. For a flashing-spot stimulus the maximum response occurs for a spot centered on the receptive field which exactly covers the receptive-field center [27]. When the spot size is gradually increased to also stimulate the inhibitory surround, the response is gradually reduced until the entire surround is also covered. This phenomenon is referred to as *center-surround suppression*, and it is known that such suppression is increased for RCs compared to the GCs that provide the dominant feedforward input [27]. A part of this increased suppression likely stems from feedforward mechanisms in the dLGN circuit, i.e., a broad feedforward retinal input to LGN interneurons, in turn providing increased feedforward surround inhibition to the RCs [27, 28]. Increased center-surround suppression implies that the neurons are less responsive to broad visual stimuli and instead more tuned to narrow stimuli or sharp spatial variations in the visual scene. Thus dynamical tuning of this suppression may be a mechanism for the nervous system to adapt to changing light conditions and viewing demands to create an efficient representation of the stimulus [29].

Although the receptive fields of dLGN cells appear largely determined by the feedforward retinogeniculate input, corticothalamic feedback has been shown to increase the inhibitory surround, i.e., increase the suppression to very large stimuli [4, 12, 13, 15, 16, 19, 30]. Other studies have reported enhanced responses of dLGN neurons [18, 30, 31] when using smaller stimuli. Interestingly, cortical feedback has been experimentally observed to increase the surround suppression both for flashing spots [32] and patch gratings [4, 19], though, the increase has been found to be larger for patch gratings [2, 4]. The topic of the present modeling study is to investigate what aspects of the thalamocortical loop, and in particular what type of cortical feedback pattern, may underlie these observed changes in RC center-surround antagonism.

While the use of computational modeling to study the effect of cortical feedback on visual processing is not new, previous projects have investigated feedback effects on the temporal processing of RCs [33–38]. Modeling studies of spatial aspects have to our knowledge been limited to relatively simple firing-rate models [39, 40] where, for example, dLGN INs have not been explicitly included. The focus in [39] was on exploring cortical feedback effects on observed effects of RC responses to discontinuity in orientations in gratings in bipartite stimuli. In [40] the *extended DOG* (eDOG) model was introduced, allowing for analytical explorations of effects of cortical feedback in certain settings, i.e., with certain combinations of excitatory and (indirect) inhibitory feedback from ON- and OFF-center cortical cells onto RCs. In that study a preliminary use-case showed that a phase-reversed (‘push-pull’) arrangement of cortical feedback where ON-center RCs receive direct excitation from OFF-driven cortical cells and balanced indirect inhibitory feedback from ON-driven cortical cells, may provide increased center-surround antagonism.

Here we instead consider a biophysically detailed model where RCs and INs, as well as orientation-selective layer-6 pyramidal cortical cells (PYs), are explicitly included. The model is an extension of a recently developed network model of the feedforward part of the dLGN circuit [41]. The neuron models include a host of Hodgkin-Huxley type active conductances [42–44], and an important feature is the multicompartment IN model that incorporates both axonal and triadic inhibition of RCs [45]. Another key element of our model circuit is the explicit incorporation of both ON-symmetry and OFF-symmetry cells which, unlike for the rate-based eDOG model [40], allows exploration of a wide range of putative synaptic patterns for the feedback from cortical cells to RCs and INs, i.e., both same symmetry (ON to ON, OFF to OFF) and cross-symmetry (ON to OFF, OFF to ON). By comparing results from a wide range of feedback patterns, we find that our results support that a phase-reversed arrangement of the cortical feedback seems most effective in increasing the center-surround antagonism observed both for flashing spots and, even more significantly, for patch gratings.

## Methods

### Overview of the network model and feedforward connections

**Fig 1:**
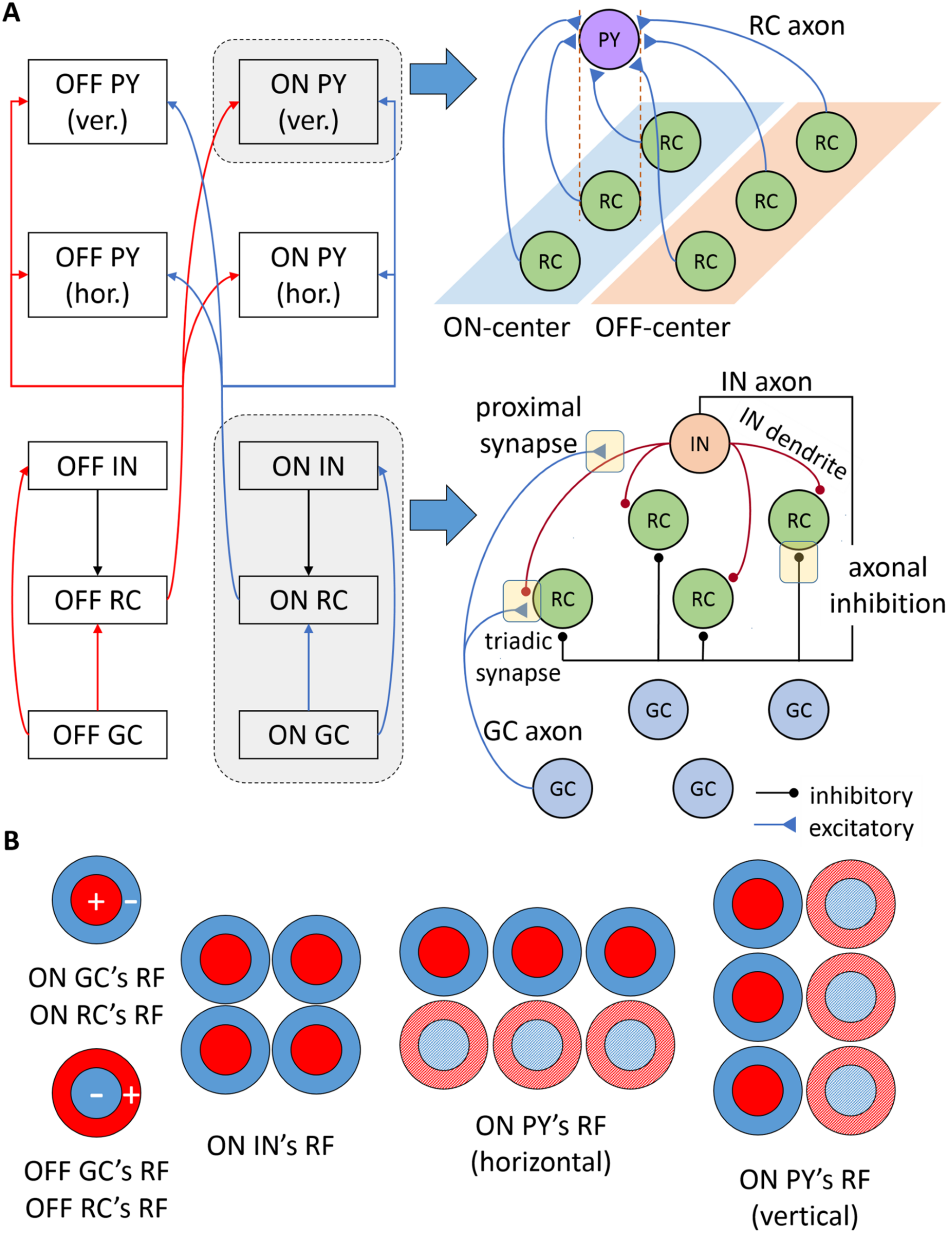
Schematic of the model circuit and feedforward connections. A: Neuronal populations and their connectivity patterns. On the left, each arrow represents synaptic connections between a source and a target population. On the right, a detailed view is shown of the spatial organization of input synapses for an ON-center PY (top) and for RCs and INs in the LGN (bottom). PYs with ON symmetry receive strong input from an elongated area of three ON-symmetry RCs and weak input from an adjacent row of three OFF-symmetry RCs. In the LGN, every IN receives input from GCs via the triadic synapse and the proximal IN dendrite. RCs are contacted by the IN axon, receiving axonal inhibition, and by the IN dendrite at the triadic synapse, resulting in direct triadic inhibition. For the sake of clarity, only excitatory connections of a single GC to the IN are shown. B: Illustration of formation of receptive fields at each stage based on the spatial arrangement of receptive fields of the synaptic afferents. RCs with ON (OFF) symmetry receive input from single ON-symmetry (OFF-symmetry) GCs with circular center-surround receptive fields. INs with ON symmetry receive input from four ON-symmetry GCs. The strong flank of the PY’s receptive field is shown in solid color and the weak flank is represented by a pattern fill. PYs both with horizontal and vertical orientation-selectivity are shown.

The core of the network model comprises two-dimensional grids of synaptically connected dLGN and cortical neurons of ON and OFF receptive-field arrangements. The network is driven by dLGN neurons that receive spikes encoding visual input from the retina. The network includes populations of retinal ganglion cells (GC), dLGN RCs and INs, and PYs of layer 6 in the primary visual cortex (Fig 1). Each layer is scaled to span a monocular patch of 10 deg × 10 deg in the visual field and contains 10 × 10 neurons of each symmetry type (ON/OFF), except in the case of dLGN INs for which there are 25 per symmetry type (20% of the total number of dLGN cells [46]). Based on the wiring rules of the cat dLGN, it has been estimated that a 1 deg × 1 deg patch of the dLGN contains about 10 RCs of one symmetry type on average at an eccentricity of 7 deg [47]. Thus, one simulated RC in our model would correspond to about 10 RCs of the cat dLGN. In the tuning of the model, we have chosen model parameters giving GC and RC responses similar to the cat experiments described in [27, 28]. Here the recordings were done on cells with receptive fields centered in areas of the visual field some distance away from the center of gaze (*area centralis* in cat).

Retinal GCs have a circularly symmetric center-surround receptive field that is inherited by dLGN RCs through one-to-one excitatory synapses as shown for cells of the ON and OFF pathways in Fig 1. In these receptive fields, the center and surround present an antagonistic push-pull arrangement [48]. A bright stimulus confined to the center of the ON-cell receptive field or a dark stimulus placed on the surround of the receptive field evoke a depolarization of the ON cell. By contrast, an ON cell is hyperpolarized by projecting either a dark stimulus to the center of the receptive field or a bright stimulus to the surround. The opposite behavior applies for OFF-center cells.

The feedforward elements of the dLGN are the same as in [41]. LGN INs receive input from four retinal ganglion cells via the triadic synapses and the proximal IN dendrites. RCs receive axonal inhibition through the IN axon and triadic inhibition by the IN dendrites at the triadic synapses, resulting in fast inhibition.

The cortical populations of PYs receive strong input from an elongated area of three RCs of the same symmetry and weak input from an adjacent row of three RCs of the opposite symmetry. PYs come in two different orientation-selectivity variants: horizontally-selective or vertically-selective. Further, each of these two cortical populations also come with ON and OFF symmetry making a total of four distinct cortical populations. This is a simplified representation of the thalamocortical loop as it neglects that the strongest thalamic input to primary visual cortex arrives in layer 4 while the feedback inputs to dLGN cells come from cells in layer 6.

The models for the dLGN and cortical neurons are all biophysically detailed in the sense that they include a variety of Hodgkin-Huxley type active conductances explicitly reproducing generation of action potentials. The GC spiking mechanism is not modeled explicitly, instead this input is modeled by means of phenomenological filter models as in [41].

### Retinal input

#### Descriptive filter model of retinal ganglion cells

The input spike trains from GCs were generated by non-stationary Poisson processes with firing rates determined by a response function *R*_g_(***r***, *t*). The response function *R*_g_(***r***, *t*) is defined as a non-separable center-surround filter that takes into account the additional delay between the center and surround signals [49–51]:

$$\begin{array}{c} R_{g}^{\text{ON}}\left(r,t\right)=H[C(r,t)-S(r,t)]. \end{array}$$ (1)

Here the response is the difference between the center signal, *C*(***r***, *t*), and the surround signal, *S*(***r***, *t*). *H*[*x*] = *xθ*(*x*) is introduced to enforce nonnegative firing rates, where *θ*(*x*) is the Heaviside step function. The difference between the center and the surround is reversed for the OFF-center ganglion cell:

$$\begin{array}{c} R_{g}^{\text{OFF}}\left(r,t\right)=H[S(r,t)-C(r,t)]. \end{array}$$ (2)

The center and surround signals are obtained by convolution between the stimulus signal, *s*(***r***, *t*), and linear spatial ($G_{a_{\text{C}}}$, $G_{a_{\text{S}}}$) and temporal ($T_{n_{\text{O}},\tau_{\text{O}}}$, $E_{n_{\text{C}},\tau_{\text{C}}}$, $E_{n_{\text{S}},\tau_{\text{S}}}$) filters:

$$C(r,t)=G_{a_{C}}\left(r\right)*T_{n_{O},\tau_{O}}\left(t\right)*E_{n_{C},\tau_{C}}\left(t\right)*s\left(r,t\right),$$ (3)

$$S(r,t)=\omega *G_{a_{S}}\left(r\right)*E_{n_{S},\tau_{S}}\left(t\right)*s\left(r,t\right).$$ (4)

Temporal filters *E*_*n*,*τ*_(*t*) are normalized low-pass filters implemented as an exponential cascade:

$$\begin{array}{c} E_{n,\tau }\left(t\right)=\frac{{\left(nt\right)}^{n}e^{-nt/\tau }}{\tau^{n+1}\left(n-1\right)!}, \end{array}$$ (5)

where *τ* is the time constant and *n* the number of low-pass filtering stages. $T_{n_{\text{O}},\tau_{\text{O}}}$ is a high-pass temporal filter that modulates the overshoot that follows the stimulus onset, observed experimentally [25, 27]. It is computed as the difference between the Dirac function, weighted by the overshoot amplification factor *β*, and a low-pass temporal filter:

$$\begin{array}{c} T_{n_{O},\tau_{O}}\left(t\right)=\beta \delta_{0}\left(t\right)-E_{n_{O},\tau_{O}}\left(t\right). \end{array}$$ (6)

Spatial filters are implemented by means of the well-known normalized Gaussian function [25, 28, 49, 50]:

$$\begin{array}{c} G_{a}\left(r\right)=\frac{1}{\pi a^{2}}e^{-r^{2}/a^{2}}, \end{array}$$ (7)

with *a* the spatial extent of the kernel. Thus, *a*_C_ defines the size of the center receptive field and *a*_S_, of the surround.

#### Visual stimuli

With the spatiotemporal stimulus function *s*(***r***, *t*) specified, the GC response can be computed by means of Eqs 1 and 2. The two main visual stimuli explored in the present work were (i) flashing circular spots and (ii) circular drifting patch gratings. In addition, separate simulations with flashed bright and dark spots within the ON and OFF subregions of different cell types were done to map out the receptive fields.

Each trial of the flashing-spot stimulus consisted of a 500 ms period of full-field isoluminant background followed by a 500 ms period in which the circular spot was superimposed on the background. The luminance profile of the flashing-spot stimulus can be described mathematically as

$$\begin{array}{c} L\left(r,t\right)=\{\begin{array}{cc} L_{\text{bkg}}\; & \text{for}\;t<500\;\text{ms},\\ L_{\text{bkg}}\left(1-\theta \left(d_{s}/2-r\right)\right)+L_{\text{stim}}\left(1-\theta \left(r-d_{s}/2\right)\right)\; & \text{for}\;500\;\text{ms}\leq t<1000\;\text{ms}, \end{array} \end{array}$$ (8)

where *d*_s_ is the diameter of the circular spot. The circular spot was concentric with the receptive field of the central GC, located in the 6th row and 6th column of the 10 × 10 grid where we set ***r*** = 0. In our formalism the stimulus *s*(***r***, *t*) is represented via an (unspecified) sigmoidal function of the luminance *L*(**r**, *t*), that is, *s*(**r**, *t*) = *l*(*L*(**r**, *t*)), where *l* is a sigmoidal *activity function* of some form, converting luminance to firing rates [28].

For the second stimulus, a circular patch of sinusoidal grating with horizontal orientation was presented for 2000 ms on a full-field isoluminant background. The luminance profile of this stimulus can mathematically be described as [40]:

$$\begin{array}{c} L\left(r,t\right)=L_{\text{bkg}}+\left(L_{\text{stim}}-L_{\text{bkg}}\right)\left(1-\theta \left(r-d_{\text{pg}}/2\right)\right)\cos\left(k_{\text{pg}}r-\omega_{\text{pg}}t\right), \end{array}$$ (9)

*k*_pg_ and *ω*_pg_ are the wave vector and the angular frequency of the patch grating, respectively, and *d*_pg_ is the diameter of the circular patch. Note that a circular spot stimulus is obtained for |***k***_pg_| = *ω*_pg_ = 0. *ν*_pg_ = |***k***_pg_|/2*π* and *f*_pg_ = *ω*_pg_/2*π* are the spatial and temporal frequencies of the patch grating, respectively. In the present applications we used *ν*_pg_ = 0.15 cycles/ deg and *f*_pg_ = 1 Hz. While the activity function *l*(*L*) could have an arbitrary sigmoidal form for the flashing-spot stimulus, it is assumed to be linear for the patch-grating stimulus, i.e., *s*(**r**, *t*) = *l*(*L*(**r**, *t*)), where *l* is of the form *l*(*x*) = *cx* for some (unspecified) constant *c* [52].

The spatial part of the convolution between the stimulus and $G_{a_{\text{C}}}$ and $G_{a_{\text{S}}}$ can be computed analytically both when the Gaussian is concentric with the spot stimulus and when it is non-concentric [28, 40]. Parameters listed in Table 1 were tuned to approximate simultaneously the spatial properties of the GC response to the experimental results obtained with a spot stimulus [27] and the temporal properties to the range of values reported by Usrey et al. [53]. The two values of *l*_bkg_ ≡ *l*(*L*_bkg_) and *l*_stim_ ≡ *l*(*L*_stim_) in Table 1 correspond to the GC response for the flashing spot (left) and the patch grating (right). A larger background level was used for the patch-grating stimulus to avoid rectification of the GC response for the negative period of the sinusoid and to assure a roughly linear GC response.

**Table 1:** Parameters of the response function of GCs. The two values of *l*_bkg_ and *l*_stim_ in the last two rows correspond to the GC response for the flashing spot (left) and the patch grating (right).

Parameter	Description	Units	Value	
*β*	Overshoot amplification factor		2.0	
*ω*	Center-surround relative strength		0.85	
*n*_O_	Filtering stages of the overshoot		1.0	
*τ*_O_	Time constant of the overshoot	ms	30.0	
*n*_C_	Filtering stages of the center signal		4.0	
*τ*_C_	Time constant of the center signal	ms	20.0	
*n*_S_	Filtering stages of the surround signal		5.0	
*τ*_S_	Time constant of the surround signal	ms	50.0	
*a*_C_	Center width	deg	0.62	
*a*_S_	Surround width	deg	1.26	
*ν*_pg_	Spatial frequency of the patch grating	cycles/ deg	0.15	
*f*_pg_	Temporal frequency of the patch grating	Hz	1.0	
*l*_bkg_(1 − *ω*)	GC response rate to the background	s^−1^	36.8	78.75
*l*_stim_(1 − *ω*)	GC response rate to the stimulus	s^−1^	56.5	89.25

### Neuron models

#### dLGN interneuron (IN)

We used the same IN and RC models as in previous work [41]. The IN model consisted of a cylindrical soma of radius 8.72 μm and length 15.3 μm, with four identical linear ‘stick’-like dendrites of length 500 μm, a set of passive membrane properties, and seven active channel conductances including the traditional Hodgkin-Huxley type sodium and potassium channels for generating action potentials, a hyperpolarization-activated cation channel, a low-threshold, T-type calcium channel, a high-threshold, L-type calcium channel, a medium-duration, calcium-dependent afterhyperpolarization channel, and a long-lasting calcium-activated non-specific cation channel. The IN model was an adapted version of the multicompartmental interneuron model in [45, 54]. For a list of the model parameters, see Table 2 in [41].

#### dLGN relay cell (RC)

RCs can be considered electronically compact [55] and thus we used a single-compartment model. The RC model corresponds to the model in [42] and includes the standard Hodgkin-Huxley type sodium and potassium channels, as well as the T-type calcium channel. While most of the parameters of the RC model are maintained as in [41], the maximal conductance of the T-type calcium channel, *g*_CaT_, was reduced to 0.2 mS/cm^2^ to force the RC to respond in the tonic firing mode even with strong disynaptic inhibition from cortical cells. For further details on the model parameters, see Table 4 in [41].

#### Cortical pyramidal cell (PY)

The thalamocortical feedback loop in mammalian brain involves more than just a single cortical population and a single cortical layer. Both neurophysiological and neuroanatomical studies have shown that layers 4 and 6 of the visual cortex are the main postsynaptic targets of the geniculate inputs and that dLGN cells receive cortical-feedback afferents only from layer 6 of the visual cortex (reviewed in [2, 4, 5]). While a monosynaptic excitatory feedback loop thalamus-cortex-thalamus involving only layer 6 is possible, intracortical processing is expected to affect the action of the thalamocortical feedback loop. Detailed modeling of this intracortical processing is beyond the scope of this work, and we instead represented the effect of cortical feedback in a reduced way by neglecting the layered organization of cortical processing (which is in accordance with other modeling approaches [33, 35, 38–40, 43]). Further, only one type of cortical neuron was included in the model, a PY.

**Fig 2:**
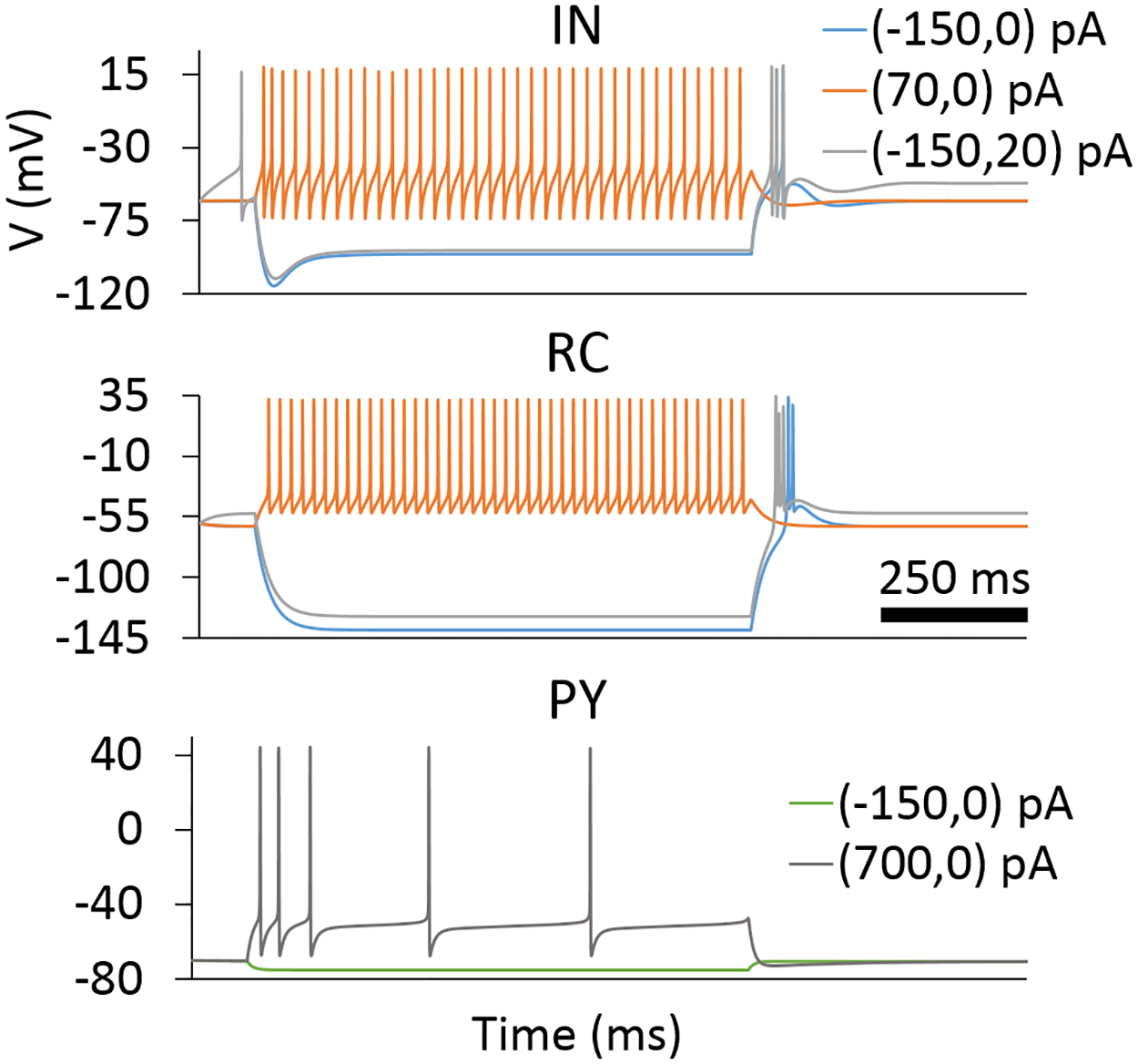
Spiking patterns of model neurons for somatic current injection. Somatic membrane potentials of the model neurons following injection of depolarizing (positive) and hyperpolarizing (negative) current steps lasting 900 ms (first of the two numbers in parenthesis). A depolarizing current step of 0 or 20 pA (second number in parenthesis) is applied afterwards.

The single-compartment model of cortical PYs was taken from [43, 44]. This model was originally developed to investigate spindle oscillations in a network of cortical neurons, thalamic reticular neurons and RCs. The model includes the classical Hodgkin-Huxley type sodium and potassium channels for action potential generation, and a slow voltage-dependent potassium channel, *I*_M_. *I*_M_ accounts for the classic ‘regular-spiking’ behavior that generates adapting trains of action potentials in response to depolarizing current injection (see Fig 2). Parameters of this cell type are summarized in Table 2.

**Table 2:** Parameters of cortical PY.

Parameter	Description	Units	Value
*A*	Neuron surface area	μm^2^	28950
*C*_m_	Membrane capacitance	μF/cm^2^	1.0
*R*_m_	Membrane resistivity	Ωcm^2^	34000
*E*_pas_	Passive leak reversal potential	mV	-70.0
*g*_Na_	Max. Na conductance	mS/cm^2^	50.0
*E*_Na_	Na reversal potential	mV	50.0
*g*_K_	Max. K conductance	mS/cm^2^	5.0
*E*_K_	K reversal potential	mV	-100.0
*g*_M_	Max. M conductance	mS/cm^2^	0.07

### Synaptic connections in the network

Conductance-based synapses were assumed, i.e.,

$$\begin{array}{c} I_{\text{syn}}\left(t\right)=wf_{\text{syn}}\left(t-t_{s}-t_{\Delta }\right)\left(V-E_{\text{syn}}\right)\theta \left(t-t_{s}-t_{\Delta }\right), \end{array}$$ (10)

for a presynaptic spike arriving at *t*_s_. Here the weight *w* is the maximal conductance of the postsynaptic receptors and *E*_syn_ is the reversal potential. *f*_syn_ is the temporal envelope of the synaptic conductance modeled as the difference between two exponential functions specified by time constants *τ*_rise_ and *τ*_decay_ (Eqs. 6.4–6.6 in [56]). *t*_Δ_ is the conduction time delay from the generation of the presynaptic spike to the initiation of the postsynaptic response and was set to a fixed value of 1 ms for all synaptic connections. Action potentials of RCs, INs and PYs were detected by upward somatic voltage crossings at −10 mV.

While AMPA receptors mediate all excitatory connections in this model, GABA_A_ receptors mediate all inhibitory synaptic interactions. Parameters of synaptic connections are shown in Table 3. Parameters of retinogeniculate and intrathalamic connections remain similar to those presented in [41]. An exception is the GC input to the IN part of the triad, for which we reduced the synaptic weight to compensate for the added excitatory input from corticothalamic connections not present in the previous model [41].

**Table 3:** Parameters of synaptic connections. Each synaptic weight *w* represents a monosynaptic connection between each source and target cell. For corticothalamic connections, instead of one synaptic weight *w*, we compared the model response for a range of different values, between the listed values.

Receptor	Location	*w* (nS)	*E*_syn_ (mV)	*τ*_rise_ (ms)	*τ*_decay_ (ms)
AMPA	GC → IN triad	0.4	10.0	0.3	2.0
AMPA	GC → IN prox.	0.3	10.0	1.6	3.6
AMPA	GC → RC	15.6	10.0	0.1	1.2
GABA_A_	IN triad → RC	6.0	-80.0	0.45	5.0
GABA_A_	IN axon → RC	4.0	-60.0	0.45	5.0
AMPA	RC^ON^ → PY^ON^	50.0	10.0	0.2	1.2
AMPA	RC^OFF^ → PY^ON^	20.0	10.0	0.2	1.2
AMPA	PY → RC	0.0–6.0	10.0	0.2	1.2
AMPA	PY → IN dend.	0.0–6.0	10.0	0.2	1.2

#### Feedforward excitation and inhibition of RCs

Following our previous network scheme [41], each GC axon synapses at two different locations, i.e., in the triadic synapse where the RC and the IN receive excitatory input, and in a proximal IN dendrite, both dependent on AMPA receptors. In particular, each GC synapses the IN in two spatially separated locations of the corresponding IN dendrite, either at the proximal dendrite (65 μm from the soma) or in the triadic synapse located distally on the dendrite (434 μm from the soma). Conversely, all four RCs are contacted by the IN axon, receiving the same GABA_A_ axonal inhibition, and by the IN dendrites at the triadic synapse, where local GABA_A_ release results in direct triadic inhibition. The triadic inhibition was modeled by means of Eq (10), and GABA release from the IN dendrites was assumed to occur whenever the local IN membrane potential at the triad crossed −10 mV (on the way upward).

#### Thalamocortical connections

Receptive fields of simple cortical cells are orientation-selective, arising primarily from oriented convergence of thalamocortical excitatory inputs of ON and OFF elongated subregions of the dLGN [48, 57–59]. On average, simple cells present two to three subregions, each with a length/width ratio of 2.5. In addition, the width of the subregion has been measured to match approximately the center of a geniculate receptive field [60, 61].

From these studies it appears that three geniculate receptive fields would be sufficient to cover one subregion of the cortical receptive field [60]. To impose such receptive fields on the cortical cells, receptive fields of model cortical PYs are formed by synaptic integration of 3 ON and 3 OFF RCs as shown in Fig 1. This minimal model of the cortical network is a base case scenario that facilitates the understanding of the key features of the circuit behavior and whose results can be easily extrapolated to more complex network architectures. Monosynaptic cortical excitation from RCs is assumed to be mediated by similar parameters of AMPA receptors as the retinal input.

#### Cortical feedback to LGN

Cortical feedback projections are mediated by a full set of cortical populations preferring different orientations [13], with a resulting net effect expected to be essentially isotropic [40]. In the model, only two orientation-selective populations are included, one preferring horizontal stimuli while the other preferring vertical stimuli.

**Fig 3:**
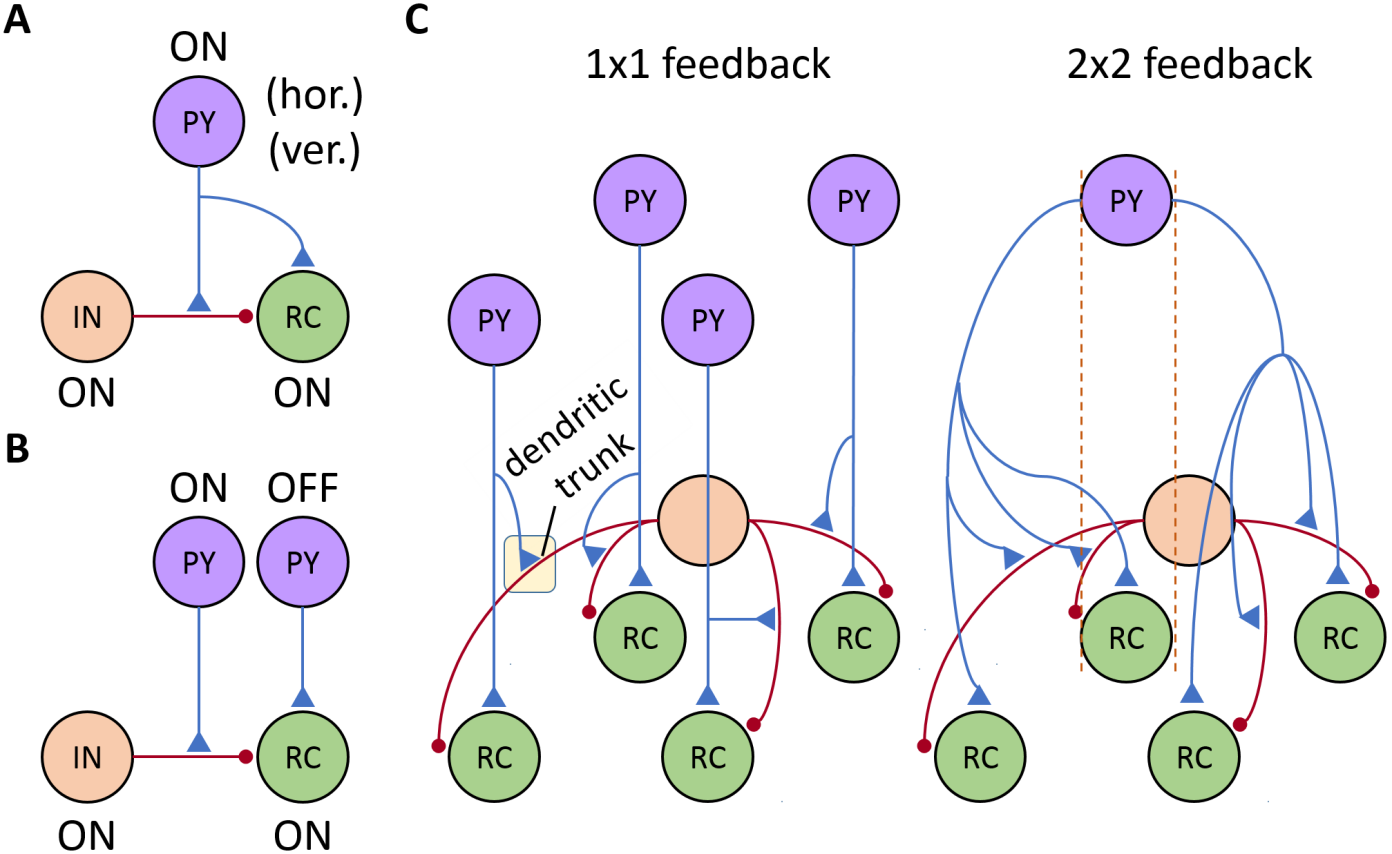
Cortical feedback configurations. Phase arrangements explored for connections between receptive fields of PYs and RCs: phase-matched (panel A) vs phase-reversed (panel B). Additionally, every RC receives feedback both from PYs with horizontal and vertical orientation-selectivity. C: The effect of the spatial spread of corticothalamic axons onto LGN cells is analyzed for another two feedback configurations: 1 × 1 and 2 × 2 cells. In the latter case, only synapses made by one PY are shown.

The detailed arrangement of the synapses of the cortical afferents in dLGN is less known, and in the present work several possible arrangements were explored (see Fig 3). In terms of spatial symmetry of receptive fields, the arrangement can be either phase-matched (A) or phase-reversed (B). With the phase-reversed feedback, effects mediated by OFF-center cortical cell drive direct excitatory input to ON-center RCs (and the opposite for ON-center cortical cells on OFF-center RCs) [62]. In the phase-matched feedback, the ON-center cortical cell synapses both ON-center INs and RCs.

For each of the two options (A,B) above, we explored two configurations for the spatial extent of the corticothalamic axon: 1 × 1 and 2 × 2 (C). In the 1 × 1 feedback, every PY synapses one spatially overlapping RC and the corresponding IN dendrite. The 1 × 1 feedback was used as a theoretical base case scenario. It has, however, been demonstrated that individual corticothalamic axons innervate an area of the dLGN that extends significantly beyond the classical receptive fiels of RCs [63]. We thus also considered a case with a spatially more widespread feedback, i.e., a 2 × 2 feedback where every PY connects an extended region of 4 RCs and the four dendrites of the IN.

The majority of retinal terminals synapse on dendritic appendages of interneurons, while cortical synapses typically establish their connections on the dendritic trunks of interneurons [64]. In the model, we placed cortical synapses on the trunk, 391 μm from the soma. With this relatively distal location, the cortical synapses could contribute to triadic inhibition of relay cells, but had a relatively minor impact on the somatic action potential generation in INs, which is predominantly driven by retinal input [64, 65].

### Simulation and analysis of results

#### Area-response curves

Area response curves were computed for two types of stimuli: flashing circular spots and patch gratings. Simulations were repeated by varying the spot diameter (patch diameter), ranging from smaller than, to larger than the receptive field center of the central RC. In particular, the spot diameter was increased from 0 to 10 degrees, every 0.2 degrees, giving a total of 51 different stimulus sizes. Raw poststimulus time histograms (PSTHs) were collected for all cells in the model. These PSTHs were averaged over 100 trials for the flashing spot and over 400 trials for the patch grating.

Averaged PSTHs were used to obtain the firing rates within specific time intervals of the stimulus. The firing rates for each stimulus diameter were used to construct the area-response curves, also known as area-summation curves [19, 27, 66]. For the flashing spot, each data point of the area-response curve corresponds to the mean firing rate computed during the second 500 ms period of the simulation [27].

A standard way to analyze the response for harmonically oscillating stimuli such as patch gratings is to compute the amplitude of the first harmonic. However, in the present simulations the duration of the patch-grating stimulus was too short to reliably compute this first-harmonic amplitude by a conventional Fourier transform. We instead estimated the amplitude of the first harmonic as follows: (1) Pick a time range corresponding to one complete period of of the oscillation. Here we chose the time period from 1000 to 2000 ms to avoid any transient effects from the upstart of the simulation. (2) Compute the DC component [67], i.e., the mean firing rate in this time window. (3) Subtract the DC component to the patch-grating response and (4) rectify the resulting signal by using the absolute value. The mean value of the rectified response over one full cycle corresponds to the mean rectified deviation of the response. For a sinusoidal modulation of the firing rate, this mean rectified deviation corresponds to 2/*π* ∼ 0.64 of the amplitude of the modulation.

Area-response curves were computed from the mean rectified deviation over one full cycle. The DC component was added to the mean rectified deviation to facilitate a visual comparison, in absolute terms, between the flashing-spot and patch-grating results. The area-response curves of both the flashing circular spots and patch gratings were filtered with a seven-point rectangular window to produce smoother curves. Additionally, the 0-degree response was added to the interpolated area-response curve to have the reference of background firing for computing the normalized response.

The normalized firing rate of area-response curves $\hat{R}\left(d\right)$ is calculated as

$$\begin{array}{c} \hat{R}\left(d\right)=\frac{R(d)-\text{min}(R(d))}{\text{max}(R(d)-\text{min}(R(d)))}, \end{array}$$ (11)

where *R*(*d*) is the unnormalized area-response. Two quantities extracted from the area-response curves are of particular interest here: the stimulus diameter giving the largest response (corresponding to the RF center size for the case of flashing spots) and the center-surround antagonism coefficient [27, 28]:

$$\begin{array}{c} \alpha =100\%\cdot \left(R_{c}-R_{\text{cs}}\right)/\left(R_{c}-R_{\text{bkg}}\right), \end{array}$$ (12)

where *R*_c_ is the maximum response, *R*_cs_ is the minimum response to spot/patch diameters larger than the receptive field center diameter, and *R*_bkg_ is the background firing rate. *α* provides a measure of the receptive-field surround suppression, where a value of 100% means that the surround suppression is strong enough to cancel out the visually-driven response to center stimulation.

#### Receptive fields

We here used two types of receptive fields: both the traditional *spike receptive field* where the trial-averaged spiking response to visual test stimuli is considered [26, 68] and the *membrane-potential receptive field* where the corresponding trial-averaged membrane-potential response is considered [48, 69].

We characterized the spike receptive fields of RCs and PYs by computing their spatiotemporal profiles (*x*-*y*-*t* receptive field maps and *x*-*t* plots) [26, 68]. The space was divided in a grid of 20 × 20 sampling points, i.e., one point every 0.25 degrees, and bright and dark spots were briefly flashed for 40 ms at every point. For every trial we ran a simulation of 300 ms where the spot was ON from 100 to 140 ms. PSTHs of the center cell (located in the 6th row and 6th column of the 10 × 10 grid) were collected and averaged across 100 trials. The spot has a diameter of 1 degree, which is the optimal stimulus size to cover the thalamic receptive field and was flashed at 50% contrast.

A composite receptive-field profile is obtained by computing the difference between the PSTHs for the bright and dark stimuli [68]. With this approach we obtained a complete *x*-*y*-*t* receptive field map over a range of values of *t*. Given that *x* is the dimension that cuts across the elongated bright- and dark-excitatory subregions, we integrated along *y* the different *x*-*y* receptive field maps at spaced time intervals (10 ms) to obtain the *x*-*t* plot. Plots were smoothed with the use of a Gaussian filter (*σ* = 1 deg) and displayed as contour plots.

To further characterize the spike receptive fields of PYs we used a measure to assess the spatial segregation of subregions within the receptive field, the *overlap index*, as described in references [48, 69]. The overlap index was computed as follows:

$$\begin{array}{c} Overlap\;index=\frac{0.5W_{p}+0.5W_{n}-D}{0.5W_{p}+0.5W_{n}+D}, \end{array}$$ (13)

where *W*_p_ and *W*_n_ are the widths of the ON and OFF subregions, respectively, and *D* is the distance between peak positions of each subregion. Values ≤ 0 denote separated subregions and values close to 1 mean symmetrically overlapped subregions. These parameters are computed from the raw *x*-*y*-*t* receptive field maps (before Gaussian smoothing) of the PY to bright and dark stimuli, within the time window from 130 to 150 ms. First, we search for the *x*-*y* positions of the peak responses to bright and dark stimuli and the corresponding value of *D*. From the peak responses, moving to the right and to the left in the *x* dimension, the two points whose responses were $1/\sqrt{e}$ of the peak response delimit the values of *W*_p_ and *W*_n_.

To characterize the mebrane-potential receptive fields of the different cells, bright and dark spots were flashed only within the center of the ON and OFF subregions of a PY and on the center of a thalamic RC. For every trial we ran a simulation of 300 ms where the spot was ON from 100 to 140 ms. In this case, somatic potentials of the center cell were collected and averaged across 100 trials. A *push-pull index*, as described in [48, 69], was used to determine the relative weight of the antagonistic response to stimuli of opposite contrast:

$$\begin{array}{c} Push\text{-}pull\;index=\frac{\left|P+N\right|}{\max(|P|,|N|)}, \end{array}$$ (14)

where *P* and *N* represent synaptic responses to the bright and the dark stimuli, respectively. Synaptic responses are defined as the average membrane potential that was above or below the baseline within a time interval centered near the peak response (also a time window from 130 to 150 ms) [48, 69]. The baseline is computed from the first 100 ms preceding stimulus onset. While the index is 0 for stimuli of opposite contrast that evoke excitatory and inhibitory responses of identical magnitude, the index is 1 for stimuli in which only one contrast generates significant responses.

#### Numerical implementation

The network model was implemented in Python using Object Oriented Programming [70], which defines a set of classes of objects describing the attributes and methods of the different neuron types and the ganglionar input. Individual cells were created with the Python package LFPy [71] that relies on the NEURON simulator [72] to compute their membrane potentials. Neurons of the network were connected by means of NetCon objects and synaptic connections simulated as discrete events [73]. In addition, we implemented an interface for creating two-dimensional layers of neurons placed in space and connecting them through topology masks. By contrast, the input spike trains from GCs were simulated using NEST 2.8.0 [74, 75] as a Poisson spike generator (*poisson_generator*).

Simulations of the model for the different stimulus sizes were parallelized in the Stallo supercomputer cluster [76] based on the MPI interface [77]. The Stallo cluster has 304 compute nodes that embed Intel Xeon E5 2670/2680 processors of 16 cpu-cores and 32/128 GB memory. We chose an MPI distributed-memory parallelization implemented with the Python library *mpi4py* [78] whereby simulation of every spot size is mapped to one MPI process. Simulation of 1 of the 51 different stimulus conditions within a trial took on average 2.4 minutes. By running 64 processes in parallel, computation of the area-response curves took 4 and 16 hours on average for the flashing spot (100 trials) and the patch grating (400 trials), respectively. We computed 16 area-response curves simultaneously by using up to 1024 processes.

## Results

The results are divided into two distinct parts. In the first part results for the feedforward part of the circuit is presented, mainly to validate the model against previous findings in the literature. The studies of the effects of cortical feedback are presented in the second part where the feedforward circuit explored in the first section is used as a starting point.

### Network response without cortical feedback

Before studying the effects of cortical feedback on the RC response specifically, we describe the feedforward response of the different cell types in the network model when the cortical feedback is deactivated, i.e., corticothalamic synapses from PYs to dLGN relay cells (RCs) and interneurons (dLGN INs) are disconnected. In this situation the RC response is driven only by excitation from its GC afferents and feedforward inhibition from INs.

#### Spike receptive fields

**Fig 4:**
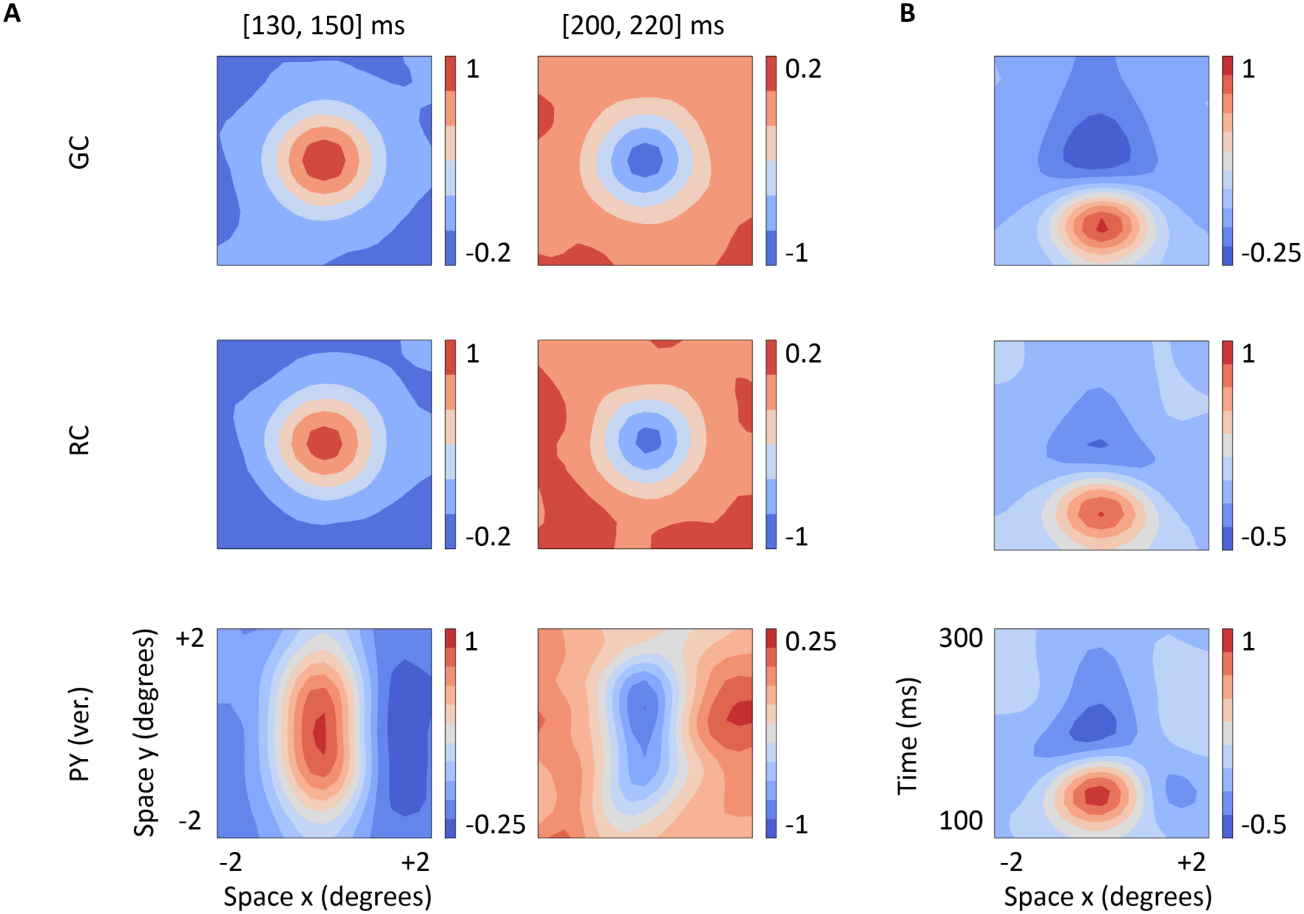
Spatiotemporal structure of spike receptive fields. *x*-*y*-*t* receptive field maps averaged over two different time windows, shortly after stimulus onset and at a later time (panel A), and spatiotemporal *x*-*t* receptive field profiles (panel B) of an ON-center GC, an ON-center RC and an ON-center vertically oriented PY. All cells correspond to the center cell (positioned at the 6th row and 6th column in 10 × 10 grid). Firing rates are normalized to the maximal firing rate. For details about computation of these receptive fields, see Methods.

The most common way to characterize response properties of cells in the early visual pathway is to measure their spike receptive fields, i.e., the trial-averaged spike response to visual test stimuli [26, 68]. In Fig 4, we show the spatiotemporal dynamics of receptive fields of cells in our network model. Panel A depicts spatial receptive field profiles at two different time intervals: one time interval centered near the peak of the center response (from 130 to 150 ms) and a second time interval centered near the minimum of the rebound decrease in the firing rate (from 200 to 220 ms). Receptive fields of GCs and RCs exhibit the characteristic properties of these cell types: circular receptive fields, with center-surround organization, and their center and surround responses are biphasic in time, consisting of an initial firing-rate increase of the center response followed by a slow rebound firing-rate decrease (the surround has a similar behavior but the respective polarities are reversed). The biphasic structure is further illustrated in the spatiotemporal *x*-*t* receptive field profiles (panel B): for *t* between 130 and 150 ms, the receptive fields of GCs and RCs show a bright-excitatory center, i.e., an increased firing to a bright spot, but for *t* larger than 200 ms, on the other hand, the polarity of the response of the receptive field center is reversed and it is seen to be dark-excitatory, i.e, increased firing-rate for dark spots.

The receptive field of the cortical cells is formed by a strong ON subregion and a weaker flanking OFF subregion. Both the center and flank subregions show also a biphasic structure in time, a feature that is inherited from the ON and OFF cells providing their afferent inputs. A visual comparison of our model receptive fields in Fig 4 with experimentally measured receptive fields shown in [26] reveals that our RC receptive field qualitatively resembles the experimental receptive field for the ‘non-lagged RC’ while our cortical-cell receptive field similarly resembles the experimental receptive field for the ‘separable simple-cell’, i.e., ON and OFF subregions are separable in the space-time domain.

From the spatial receptive field maps of the PY to bright and dark stimuli (before calculating the composite receptive-field profiles shown in Fig 4A), we estimated the widths of the ON and OFF subregions, *W*_p_ and *W*_n_, and the distance *D* between peak positions of each subregion. The position of the peak response to the bright stimulus was situated at (0, 0) degrees and the position of the peak response to the dark stimulus was at (1.25, 0) degrees, providing a distance *D* of 1.25 degrees. The widths of the ON and OFF subregions were nearly identical (*W*_p_ = *W*_n_ ≃ 1.3 degrees), as expected from the symmetrical design of the network. The overlap index was 0.02 (see Eq 13), a value that is within the range of values of cells quantitatively described as simple cells according to their membrane-potential receptive fields [48, 69].

#### Membrane-potential receptive fields

**Fig 5:**
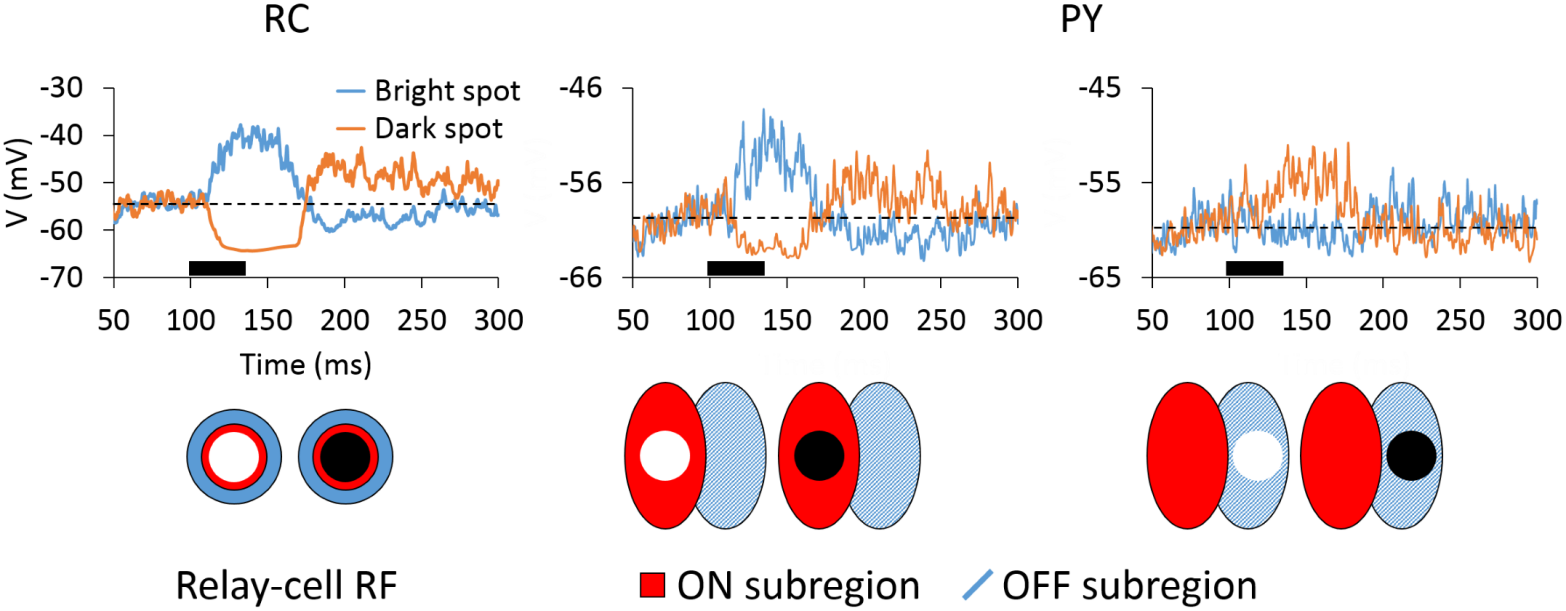
Membrane-potential receptive fields of RCs and PYs to bright and dark stimulation. Averaged somatic membrane potentials over 100 trials to bright or dark spots flashed in the receptive-field center of a RC (left) or within the ON (center) or OFF (right) subregions of a PY cell. Both cells are ON-center cells. Dashed lines indicate baseline computed from the first 100 ms preceding stimulus onset. The thick bar under the traces marks the time the stimulus is on (from 100 to 140 ms).

To further illustrate the structure of receptive fields and the antagonism between ON and OFF inputs, we show in Fig 5 the membrane-potential receptive fields of RCs and PYs to bright and dark spots, i.e., trial-averaged membrane-potential responses to bright and dark spots [79]. The push-pull effect (in terms of stimulus response) is observed both for the RC and in the different subregions of the PY, that is, stimuli of the reverse contrast evoke responses of the opposite sign. When positioned both in the receptive-field center of the ON-center RC (left panel) and in the ON subregion of the ON-center PY (center panel), a bright spot evoked a strong depolarization followed by a rebound hyperpolarization while a dark spot evoked pronounced hyperpolarization followed by rebound depolarization. The responses when stimulating the OFF subregion of the present cortical cell (right panel), were much weaker. However, as for the stimulation of the ON subregion, a push-pull pattern was observed here as well, although of opposite polarity. We also noted that the trial-averaged membrane-potential traces for the PY in Fig 5 show a higher variance because they integrate synaptic inputs from a larger pool of neurons than RCs do. Further, the presently used test spot is a suboptimal stimuli for PY receptive fields, and thus does not evoke responses as strong as for the RC.

To compare our model responses with experimental results we computed another measure, the push-pull index (see Eq 14), used previously to determine the relative weight of the antagonistic response to stimuli of opposite contrast [69]. For our model, the push-pull index was found to be 0.32 for the RC and 0.68 for the PY. The observation of a larger push-pull index for PYs compared to RCs is in general accordance with the findings of [69] (cf. Fig 4 therein), and a push-pull index of PYs between 0.6 and 0.7 is also seen for some simple cells (though here a large variation is observed in the experiments). While a comprehensive comparison with experiments is prohibited by lack of experimental data, as well as the simplistic description of cortical circuitry, we conclude that the feedforward aspects of our model circuit appear to produce plausible receptive fields.

#### Temporal response to flashing spots and patch gratings

**Fig 6:**
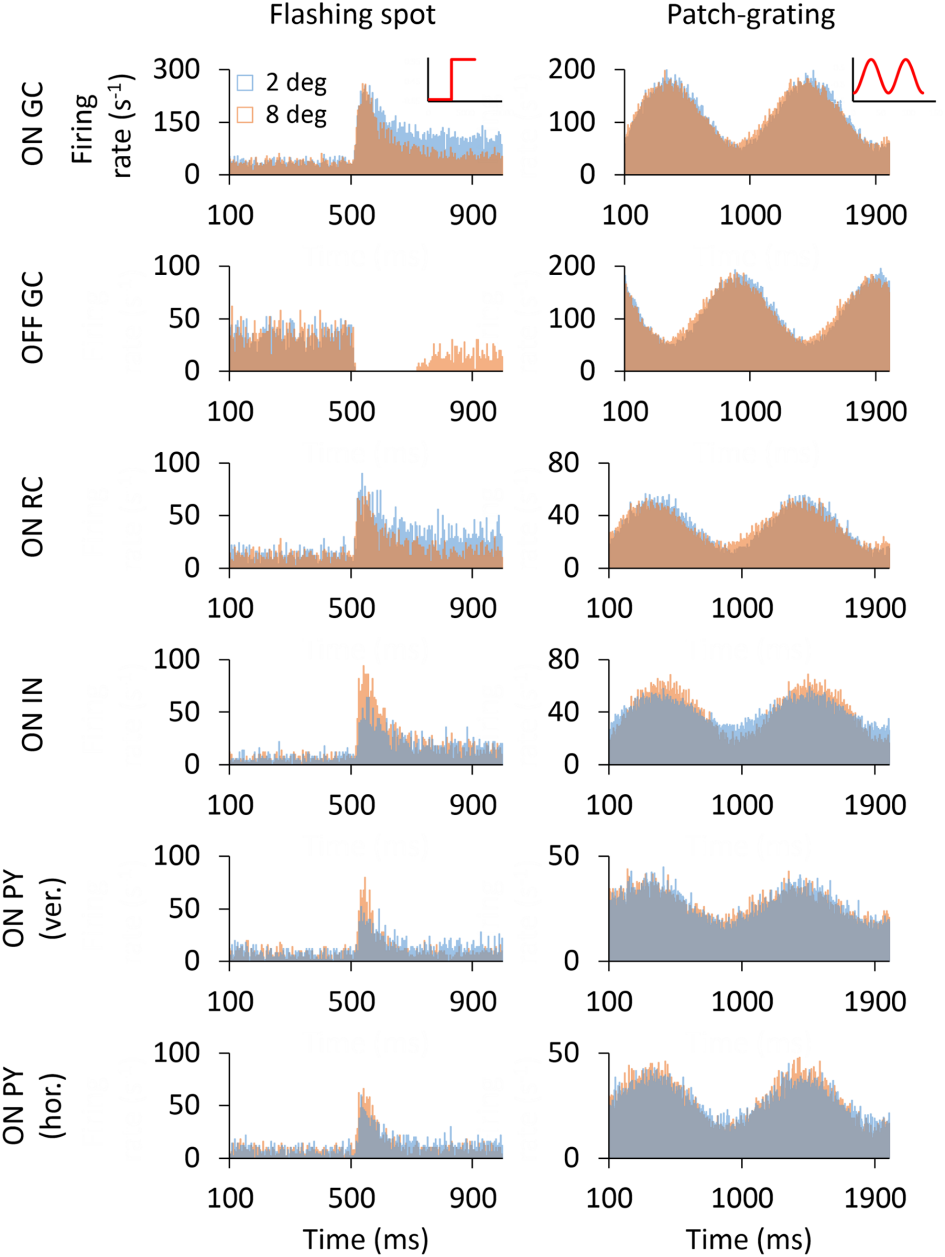
PSTHs of cells for the flashing spot and patch grating. Trial-averaged PSTHs of ON- and OFF-center GCs, ON-center RC, ON-center IN and ON-center vertically and horizontally oriented PYs for two spot/patch diameters: 2 and 8 degrees. All cells correspond to the center cells (6th row, 6th column) in the two-dimensional grids. The 8-degree responses of the IN and PYs are plotted in the front of graph for better visualization.

We next explored the temporal response of the model to flashing spots and patch gratings. Fig 6 shows the trial-averaged poststimulus time histograms (PSTHs) for cells at the center of the grid stimulated by concentric flashing spots (left column) or patch gratings (right column). For the ON GC response to flashing spots we observe similar overshoot responses to the stimulus onset for the two spot sizes considered, i.e., the 2-degree spot, which essentially covers the receptive-field center, and the 8-degree spot also covering the surround (Fig 6A).

However, the surround-inhibition evoked by the 8-degree spot substantially reduced the response after stimulus onset, resulting in a more pronounced exponential-like decay of the ON GC as observed experimentally [27]. The response of the OFF GC is suppressed for the entire time the flashing spot is on for the 2-degree spot, while for the 8-degree spot some firing is seen after approximately 200 ms.

The RC response is qualitatively similar to the response of the ON GC but the overall firing rate is lower in accordance with the lower retinogeniculate transfer ratio of the firing rate reported in [27]. The overshoot responses of the IN and PYs to the flashing spot were more pronounced for a 8-degree spot because this stimulus size better stimulates their receptive fields during the transient response. As for the GC response, the RC response reached a steady state after an exponential-like decay.

Inspection of the patch-grating responses in the right column of Fig 6 reveals that the response, i.e., amplitude modulation, to the 2-degree patch is slightly larger than the response to the 8-degree patch for both the ON and OFF GCs, as well as the ON RC. More noticeable differences were observed between responses to 2-degree and 8-degree patches when choosing smaller values of the spatial frequency *ν*_pg_ of the patch grating (see Eq 9). However, the spatial frequency selected in this work evokes smaller surround suppression in the GC response and thus facilitates the study of cortical-feedback induced effects of the increase in the surround suppression in RCs. Another noteworthy feature of both the GC and RC responses are that the 2-degree response is seen to be slightly phase-delayed compared to the 8-degree response.

For the ON IN the patch-grating results are similar to that observed with the flashing spot: there is a significant increase of the firing rate for the largest patch diameter. However, unlike for GCs and RCs, the 8-degree response is seen to be slightly phase-delayed compared to the 2-degree response. This reflects the spatial arrangement of synaptic inputs from GCs to the IN.

For PYs, an interesting difference is seen between responses of the horizontally-selective and vertically-selective cells. While the 8-degree response was substantially larger than the 2-degree response when the stripe orientation matched the orientation selectivity (horizontally-selective PY), this difference was barely noticeable when they were non-matched (vertically-selective cells PY).

#### Two-dimensional spatial representation of the network response

**Fig 7:**
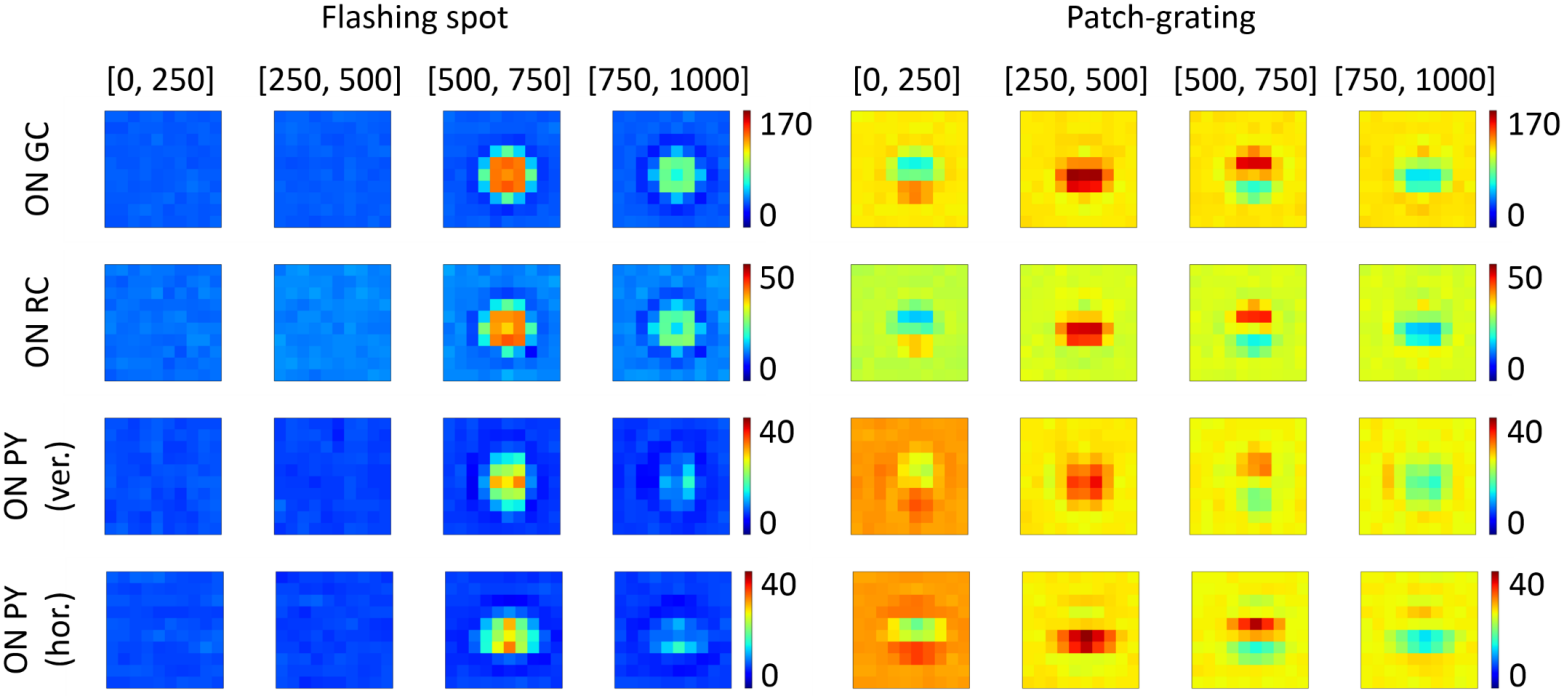
Time-averaged topographic representation of responses of cells in the network grids. Two-dimensional representations of time-averaged PSTHs of ON-center GCs, RCs and horizontally- and vertically-selective PYs. Four leftmost columns: Averaged PSTHs for the flashing-spot stimulus at four different time windows as indicated (in ms). Four rightmost columns: Averaged PSTHs for the patch-grating stimulus at the same time windows. A pixel in every panel represents time-averaged activity of one cell in the corresponding 10 × 10 grid. Color bars include values of the time-averaged firing rates. The stimuli are centered on the cell at the 6th row and 6th column of the 10 × 10 grid, and the stimulus diameter is 4 degrees.

The spatial profile of the network response is depicted in Fig 7 for the various cell types in the model. Here each color panel shows a topographic representation that includes the activity of every cell in the corresponding 10 × 10 grid, averaged across four different time intervals.

The four leftmost columns of plots show flashing-spot responses. Following the spot onset at 500 ms, the response of GCs and RCs covering the spot area reproduce the overshoot response seen in experiments [25, 27, 80], reflected in an increase of the activity that progressively diminishes and reaches a steady state for the last time interval (from 750 to 1000 ms). In the response of GCs and RCs after spot onset, we can observe the clear effects of the center-surround antagonism of their receptive fields. Thus, cells at the edge of the spot, whose receptive-field center is covered by the stimulus while a significant portion of the receptive-field surround is not, show a larger response than cells situated in the stimulus center. The response drops below the background firing for those cells whose receptive field lies just beyond the edge of the spot, producing a dark annulus of reduced activity surrounding the stimulating spot. The spatial pattern of the flashing-spot response for the RCs is qualitatively similar to that of the GCs, but the firing rates are generally smaller (similar to what was seen in the PSTHs depicted in Fig 6). The spatial profile of the flashing-spot responses of the PYs resembled those of RCs, but the orientation selectivity of the PYs has some notable effects: the horizontally selective population enhances horizontal edges of the spot stimulus while the vertically selective population enhances vertical edges (see, for example, activity of the four cells flanking the cell situated in the center of the grating).

The four rightmost columns of plots in Fig 7 show the responses to a patch grating for one complete oscillatory cycle. The circular patch contains a horizontal-striped sine wave grating moving upward. For this stimulus one expects the horizontally selective cortical neurons (PY hor.) to respond more vigorously than the vertically selective population (PY ver.). This is also what is observed: compare, for example, responses of the center cells of the horizontally-selective and the vertically-selective populations for the period between 250 and 500 ms.

#### Area-response curves

The measurement of the so-called area-response curves has been a common way to quantify the spatial response properties of cells in the early visual pathway, in particular for LGN RCs [4, 18, 19, 27, 81]. Here the response to circular spots (patches) centered on the receptive-field center is measured as a function of the spot (patch) diameter. Of particular interest for the present study is the area-response curves measured for LGN RCs since the main focus is on the role of cortical feedback on the RC response. Interestingly, the measured RC area-response curves have been observed in experiments to depend on whether cortical feedback is present or not [4]. A key goal of the present modeling study is to investigate possible mechanisms for these observed differences. Most of the following results are thus focused on such area-response curves, in particular on specific features of these curves such as the stimulus diameter giving the maximum response (corresponding to the receptive-field center size in the case of flashing spots) and the center-surround antagonism coefficient *α* defined in Eq 12.

**Fig 8:**
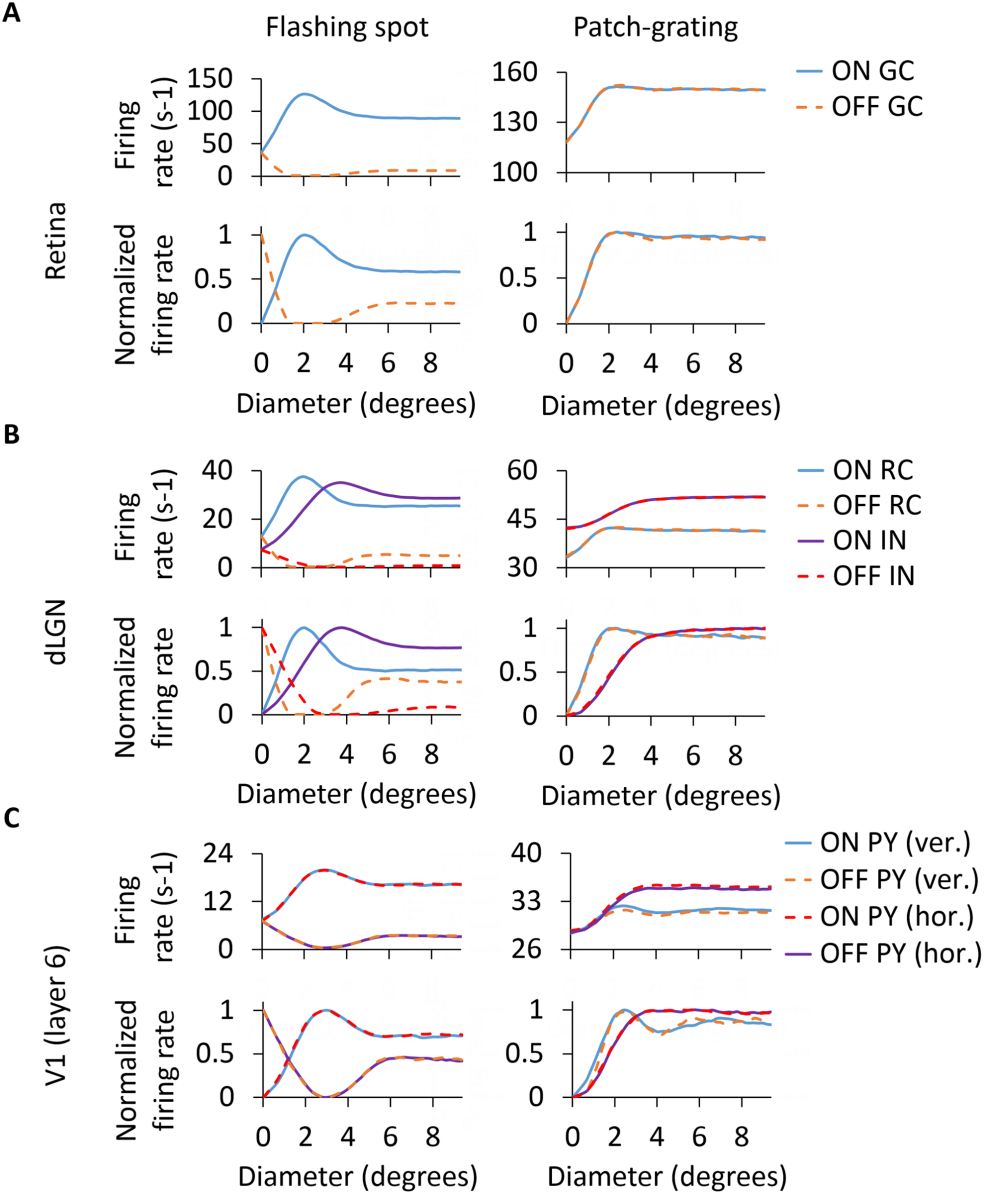
Area-response curves of cells in the model circuit including only feedforward connections. Area-response curves of GCs (A), RCs and INs (B) and PYs with horizontal and vertical orientation-selectivity (C) for the flashing spot and patch grating. For the flashing spot, each data point of the area-response curve represents the mean firing rate computed during the second 500 ms period of the simulation. For the patch grating, each data point corresponds to the mean firing rate of the rectified response over one full cycle (see Methods for further details). ON and OFF responses for the patch grating are 180 degrees out of phase.

Fig 8 shows area-response curves for the different types of cells in our model circuit. Panel A (left column) shows results for GCs, both ON and OFF cells, when bright flashing spots are used as stimuli. For the ON cell, the area-response curve reaches a maximum for a spot diameter of about 2 degrees, corresponding to the size of the receptive-field center [27, 28]. For this diameter the stimulus spot fits the excitatory center exactly. For larger spots the stimulus also covers part of the inhibitory surround, and the response is correspondingly reduced. When the spot diameter increases beyond also the surround, the response no longer changes with diameter, i.e., the area-response curve reaches a plateau level. For the OFF GC, the area-response curve has instead a dip for spot sizes similar to the receptive-field center but the response recovers when the spot diameter is increased to cover also part of the now excitatory surround.

In Fig 8B (left column), area-response curves for dLGN cells are depicted for the flashing-spot stimulus. While the magnitude of the firing rate is much reduced, the RC area-response curve qualitatively resembles that of the ON GC (panel A) that provides the feedforward excitatory input. The patch size giving the maximum response is, for example, very close to that of its retinal afferent, i.e., about 2 degrees. However, we observe a larger center-surround antagonism for the RC compared to the GC that feeds into it, i.e., the center-surround antagonism coefficient *α* is increased from 41.8% to 50.1% (see Table 4). As there are only feedforward connections in this configuration of the dLGN model circuit, the larger center-surround antagonism of the RC compared to the GC is due the feedforward inhibition via INs [41].

**Table 4:** Response measures for GC and RC for example area-response curves for flashing spots and patch gratings. *d*_c_ is the spot size giving the largest response (and corresponds to the receptive-field center size for flashing spots). The center-surround antagonism *α* is defined in Eq 12. Results from upper two rows are extracted from Fig 8. Results from the third, fourth and fifth rows are extracted from Figs 10 and 14, respectively.

	Flashing spot		Patch grating	
	*d*_c_ (deg)	*α* (%)	*d*_c_ (deg)	*α* (%)
GC	2.0	41.8	2.4	6.6
RC (without feedback)	2.0	50.1	2.4	11.2
RC (phase-reversed feedback)	1.8	61.6	2.0	26.0
RC (phase-matched feedback, Fig 14A)	2.0	49.1	2.4	11.3
RC (phase-matched feedback, Fig 14B)	2.0	54.6	2.0	18.3

The flashing-spot area-response curves of INs in Fig 8B are distinguished from the RC curves by the much larger receptive-field center diameter, i.e., about 4 degrees rather than 2 degrees. This reflects the spatially more widespread feedforward input from four neighboring GCs. We also observe a much reduced center-surround antagonism compared to RCs, in accordance with previous experimental [82] and modeling studies [28, 41]. The flashing-spot curves for the PY in panel C exhibit similar qualitative features of the INs: larger receptive-field center sizes (about 3 degrees) and smaller center-surround antagonism than for the RC. We also observe essentially identical area-response curves for the horizontally and vertically-selective PYs, reflecting the circular symmetry of the flashing-spot stimulus.

Panels in the right column of Fig 8 show the area-response curves for patch gratings. For the ON-center GC response (panel A), the shape of the curve follows the same tendency as shown for the flashing spot, in which the maximal response is seen at about 2 degrees and the response is reduced for larger diameters. However, this reduction of the response is less pronounced for the patch-grating stimulus as observed experimentally in decorticate conditions where only feedforward inputs are present like here [4].

Note, also, that the shape of the patch-grating curve depends on the chosen value of the spatial frequency *ν*_pg_ of the grating. With a lower spatial frequency than the one used here (0.15 cycles/deg; cf. Table 1), i.e., thicker grating stripes, the area-response curves would be more similar to the flashing-spot curves. The patch-grating curves for the RC in panel B also show a maximum at around 2 degrees and the center-surround antagonism *α* is increased compared to the GC curve, from 6.6% to 11.2% (see Table 4). It should be noted that the patch-grating response of OFF-center cells is 180 degrees out of phase compared to the response of ON-center cells.

The IN area-response curve for the patch grating does not exhibit a clear maximum, but is instead monotonously increasing even for patch diameters beyond the IN receptive-field center size of about 4 degrees. The patch-grating area-response curves of the PYs are shown in Fig 8C. Unlike for the flashing-spot stimulus, the response to the grating is different for the horizontally-oriented and vertically-oriented PYs when the diameter of the patch is greater than 2 degrees. While the receptive field of the horizontally-oriented PY perfectly matches the horizontal stripes of the stimulus, the receptive field of the vertically-oriented PY is perpendicular to the grating stripes, resulting in a lower firing rate as shown for the area-response curves in panel C.

### Effects of cortical feedback on the RC response

After exploring above the feedforward response of the different cell types in the network model, we now move on to investigate how cortical feedback to the dLGN circuit affects the spatial response properties of RC cells. This will depend on the detailed corticothalamic connectivity pattern which is not yet experimentally fully resolved. In the next sections, we thus present simulation results for the different alternatives considered in Fig 3.

#### Functional organization schemes of the feedback signal

**Fig 9:**
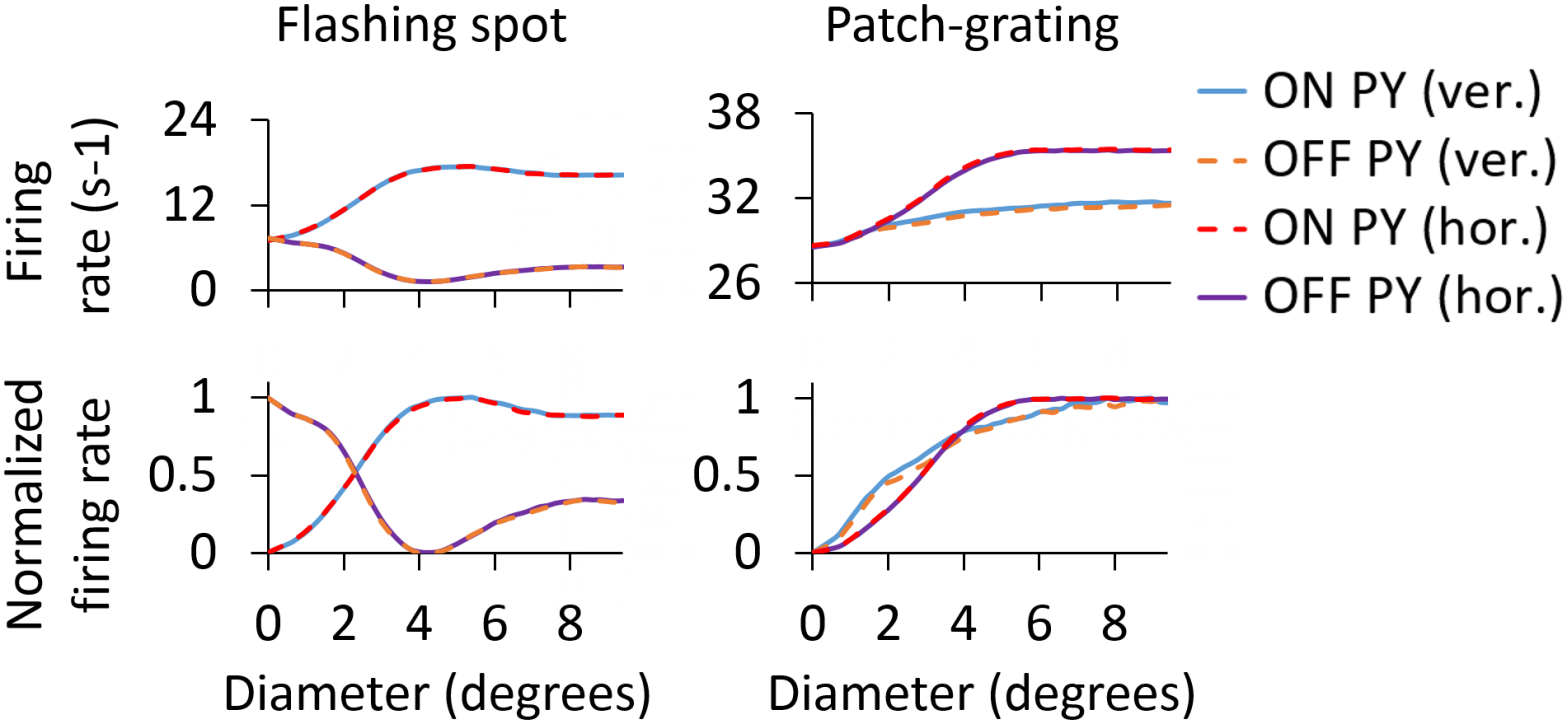
Aggregate corticothalamic signals from ON- and OFF-center PYs. ON PY (hor.) and ON PY (ver.) are the aggregate area-response curves of the 4 horizontally oriented and the 4 vertically oriented ON-center PYs, respectively, that feedback to one RC (and one dendrite of the IN) in the 2 × 2 configuration of Fig 3C. The same notation is used for the OFF-center cells.

One key aspect of the feedback signal is its spatial organization, i.e., whether each PY only feeds back to a single RC (1 × 1 configuration) or whether each PY feeds back to a cluster of four neighboring RCs (2 × 2 configuration), see Fig 3C. For the 1 × 1 case a qualitative account of the effect of the corticothalamic feedback on the RC area-response curves is obtained from the PY curves in Fig 8C, while for the 2 × 2 feedback the aggregate activity of the four PYs connected to a RC is more relevant. In Fig 9 we show this aggregate response computed by averaging the area-response curves of the four PYs in every cortical population that connect to the same RC: ON and OFF vertically-oriented, and ON and OFF horizontally-oriented. Comparison of the area-response curves for single PYs in Fig 8C, and the corresponding curves for the aggregate PY responses in Fig 9 reveals similar characteristics. As expected, the area-response curves for aggregate feedback is independent of orientation selectivity for flashing spots, both for ON and OFF cells, but not for the patch grating.

For the flashing-spot curves a difference between the aggregate area-response curves in Fig 9 and the area-response curves for single PYs in Fig 8 is that the maximum occurs for a larger spot diameter for the aggregate. Likewise, for the patch-grating curves, the plateau level is reached for a larger patch size for the aggregate. As shown below, these differences between the feedback curves from single PYs and 2 × 2 PYs for large stimulus diameters are key for understanding how the different spatial feedback configurations affect the RC response.

Another key aspect of the cortical feedback is the functional organization of synapses between ON and OFF PYs and their target ON and OFF RCs. We consider two different configurations: (i) ON PYs excite both ON RCs and ON INs (*phase-matched feedback*, Fig 3A) and (ii) OFF PYs excite ON RCs (*phase-reversed feedback*, Fig 3B). The latter arrangement implies that there is a phase-reversed *push-pull* influence from layer 6 simple cells providing feedback to the LGN, a set-up that has received support both in experimental [62] and modeling studies [40]. In analogy, the phase-matched arrangement can be also called *push-push* feedback [48, 69].

#### Phase-reversed (push-pull) cortical feedback

Corticothalamic feedback has been observed to exert both excitatory and inhibitory influences on RCs [1, 2, 4, 83]. By itself, direct excitatory feedback to RCs will generally increase the RC firing rate, while indirect inhibitory feedback via INs will reduce the firing rate. In the present model study we are particularly interested in regimes with a rough balance between excitatory and inhibitory effects so that the main effect of cortical feedback is the change in the *shapes* of the area-response curves, not their overall magnitude. Further, the model set-up and parameters values are chosen so that the IN firing rate is only modestly altered [64], i.e., all inhibitory effects from cortical feedback are mediated via triadic action from INs to RCs with little effect on IN firing. This is in accordance with the idea that the corticothalamic pathway is primarily modulatory rather than driving in nature [5]. Although we focus on ON-center RCs, a similar behavior is found for OFF-center RCs.

**Fig 10:**
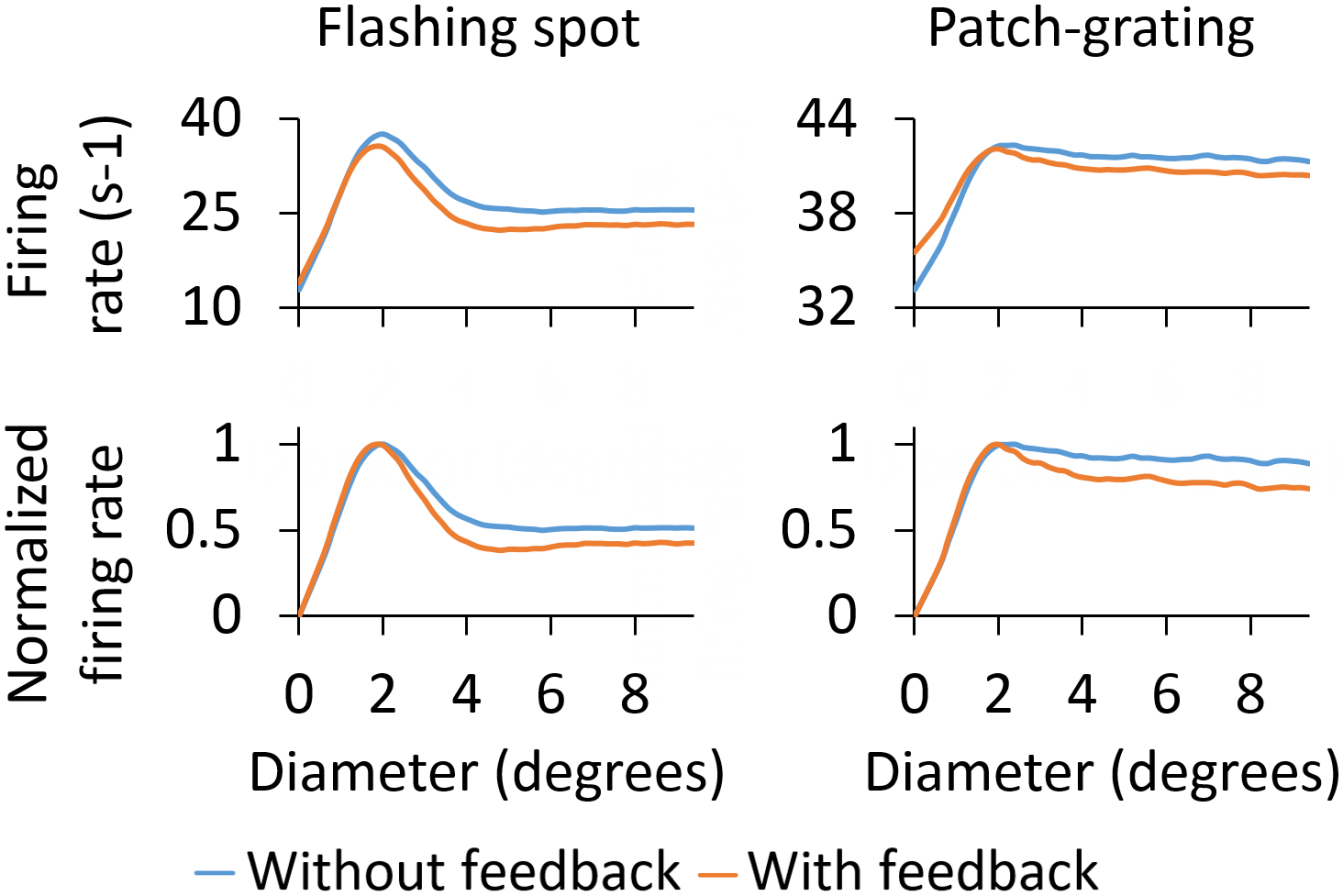
Area-response curves for ON-center RC with and without cortical feedback for the phase-reversed configuration. The RC receives feedback from a set of 2 × 2 PYs. The synaptic weight of the monosynaptic connection between PYs and RCs is set to 1.5 nS and to 0.3 nS between PYs and INs. Other parameters are listed in Tables 1–3.

*Area-response curves:* In Fig 10 we compare the RC responses with and without cortical feedback (absolute firing rates on top, normalized firing rates below). Firstly, we describe the inhibitory effects of cortical feedback on the RC response. As observed for both stimuli, the major effect is that cortical feedback increases surround suppression on RCs, which results in a reduction of the response for stimulus diameters larger than 2 degrees. The center-surround antagonism *α* is increased compared to the RC curve without feedback, from 50.1% to 61.6% for the flashing spot and from 11.2% to 26.0% for the patch grating (see Table 4).

The additional surround suppression is seen to have the signature of the aggregate response of ON-center PYs (shown in Fig 9) that feed back to the IN. Indeed, the flashing-spot response in Fig 10 has a dip for a spot size similar to the maximum of the ON-center aggregate response in Fig 9 (at about 5 degrees). Likewise, the continuous decrease of the patch-grating response with increasing patch sizes is seen to match the corresponding increase in the patch-grating aggregate response. In addition, the effectively reduced excitatory feedback from OFF-center PYs (since this feedback is out of phase with the ON-center RC) for large spot sizes (Fig 9) may also contribute to the increased center-surround antagonism.

The increase of surround suppression has been observed in experiments with both flashing spots [32] and patch gratings [4, 19], although the effect seems to be less prominent with flashing spots [2, 4]. Our example results in Fig 10 also show a larger increase of surround suppression for the patch-grating stimulus, but not as prominent as the increase reported in [4].

As seen below where the dependency of model behavior on the corticothalamic synapse weights are systematically explored, the amount of suppression and center-surround antagonism vary with model parameters. However, a general trend is that cortical feedback appears more effective in increasing surround suppression for patch gratings than for flashing spots.

Unlike for the flashing spot, cortical feedback also amplifies the patch-grating response for the smallest patches, i.e., for diameters smaller than the diameter of the receptive-field center (upper right panel in Fig 10). Thus, in this stimulus range, the competition between excitatory feedback from OFF PYs and inhibitory feedback from ON PYs results in a net excitation. This model prediction is in accordance with experimental observations of patch-grating responses on primate LGN RCs [18].

The third effect produced by cortical feedback is the reduction of the stimulus diameter giving the maximum response. For the example results in Fig 10, close inspection reveals that the size of this maximum-response diameter is slightly reduced from 2.0 degrees without feedback to 1.8 degrees with feedback for the flashing spot (where this maximum-response diameter corresponds to the receptive-field center size [27]). For the patch grating the maximum-response diameter is reduced from 2.4 to 2.0 degrees by the cortical feedback (Table 4).

**Fig 11:**
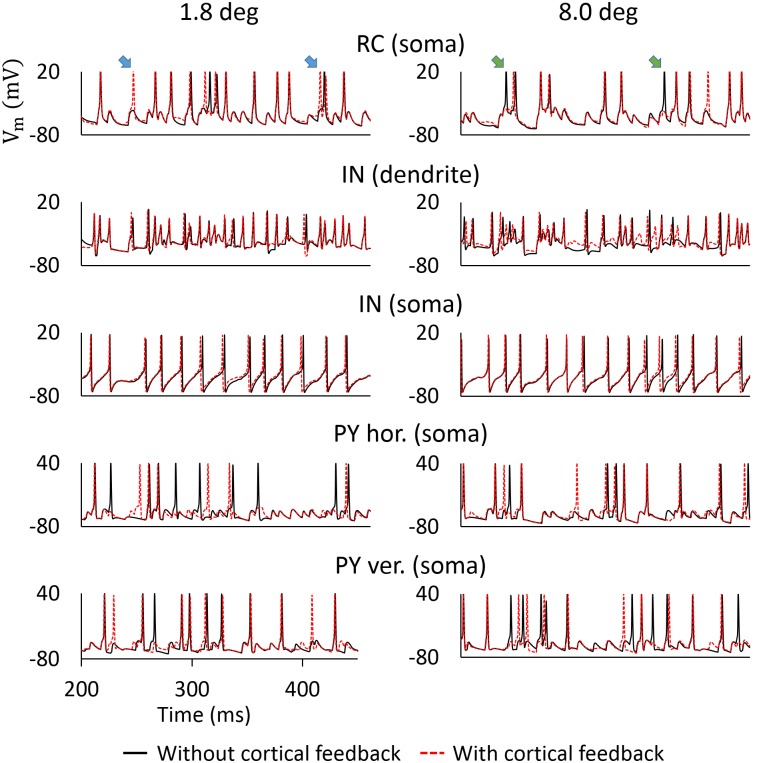
Membrane-potential dynamics for the patch grating at different stimulus sizes with the phase-reversed feedback. Somatic records correspond to ON cells situated at the center of their corresponding layers. The LGN IN dendrite depicted is the dendrite connected with the RC. Blue and green arrows indicate extra action potentials and suppression of action potentials elicited by cortical feedback, respectively. Corticothalamic synapse weights are the same as in Fig 10.

*Membrane potentials:* We next turn to an exploration of the mechanisms behind the effects observed for cortical feedback in the phase-reversed situation in Fig 10. In Fig 11 we show excerpts of membrane potentials for various cells in the model circuit with patch-grating stimulation. Two different patch diameters are considered, 1.8 degrees and 8 degrees. We note that, as the RC voltage dynamics is dominated by the driving input from retina, it was close to identical in the cases without (black lines) and with (red lines) cortical feedback included. However, exceptions to this occurred when the membrane potential was close to the action-potential threshold. At such instances, cortical input could either act to push (otherwise) subthreshold events in RCs above the threshold by providing direct excitation, or to prohibit threshold crossings (that would otherwise occur) by providing indirect inhibition of RCs via INs. Thus, the cortical feedback on RC responses is to either add or remove spikes, in accordance with previous experimental observations [34].

In the case of the small spot (1.8 degrees) the excitatory component of the cortical feedback dominated. Cortical feedback then acted to push occasional subthreshold events above the threshold, and lead to extra action potentials being fired by RCs (blue arrows in Fig 11). This explains the observation in Fig 10 that cortical feedback increased the response for patch sizes smaller than 2 degrees.

With a larger spot (8 degrees), a larger number of PY cells were activated, the spatially extended INs thus received more cortical feedback (cf., Fig 3C). Then, the inhibitory component of cortical feedback became more pronounced. The inhibitory mechanisms are complex, as INs provide inhibition both via dendodendritic and axonal GABA-release. A close investigation of Fig 11 indicates that cortical feedback predominantly acted on INs by modulating the timing (i.e., the spikes occurred earlier), rather than the amount (i.e., the number of spikes was the same) of inhibitory output. Since RCs and INs receive joint retinal input, the timing is important, and especially so in the triadic synapses, where excitation and inhibition of RCs tend to follow with a time-locked delay [84]. As Fig 11 indicates, an earlier occurrence of inhibitory output from INs tended to prevent threshold crossings in RCs (green arrows), while the opposite occurred more rarely. Therefore, cortical feedback leads to a reduced RC firing rate. This explains the observation in Fig 10 that cortical feedback increased surround suppression for large patches.

**Fig 12:**
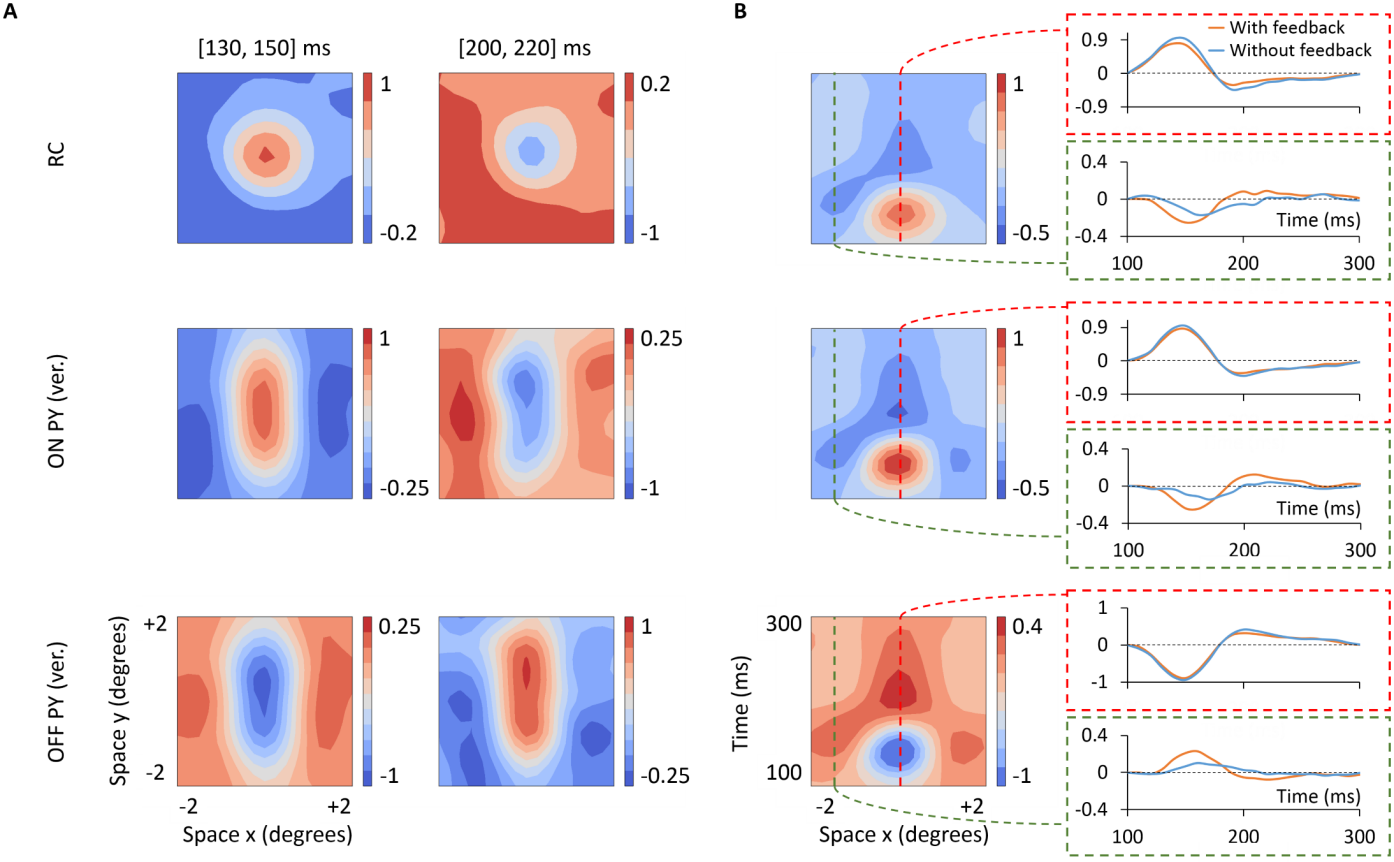
Spike receptive fields with phase-reversed cortical feedback. *x*-*y*-*t* receptive field maps averaged over two different time windows (panel A) and spatiotemporal *x*-*t* receptive field profiles (panel B) of an ON-center RC and two vertically oriented PYs of the ON and OFF-center type, respectively. The additional column on the right of panel B shows 1D temporal profiles extracted from two locations of the *x*-*t* receptive field (see Methods), one corresponding to the receptive-field center, the other two the receptive-field surround. Firing rates are normalized by the same values used in Fig 4. Corticothalamic synapse weights are the same as in Fig 10.

*Receptive fields:* We next examined effects of the phase-reversed feedback on properties of the spike receptive fields. Spatial *x*-*y*-*t* receptive-field representations and spatiotemporal *x*-*t* receptive field profiles, analogous to Fig 4 for the case without cortical feedback, are shown in Fig 12. Overall, the receptive field of the depicted ON-center RC (panel A) maintains the same spatial structure as seen for the feedforward situation in Fig 4: roughly circular receptive fields with center and surrounds both responding biphasically in time. The most striking differences between the configurations with and without feedback arise in the relative amplitudes of the center and surround responses. To better illustrate this, we have added a column on the right in panel B showing the time evolution of the center and surround responses. As seen here, cortical feedback reduces the RC center response and increases the RC surround response (in terms of the absolute value of deviation from the background firing activity).

These changes in the center response of RCs can be explained by considering the time plots of center responses of ON-center and OFF-center PYs (center and bottom right panels of Fig 12). The center response of ON PYs increases its firing rate immediately after stimulus onset, which implies increased disynaptic inhibition (mediated by LGN interneurons) and thus a reduced RC firing rate in the first part of the biphasic response.

OFF-center PYs, which give excitatory input to RCs in this phase-reversed configuration, instead decrease their firing rate in the first phase of a center-stimulus response. This further contributes to the reduced RC response to a center stimulus in the first phase of the biphasic response. For the second phase the situation is opposite. Here the OFF-center PYs increase their firing rate with a center-stimulus response, further contributing to the increased response of the RC in the second phase.

The effect of cortical feedback on the RC surround response is seen to not only quantitatively change the amplitude of the response: here the feedback is seen to provide a substantial dip in the RC firing rate for the first part of the response (up until about 180 ms, cf. second panel in the right row of Fig 12). Note that this effect cannot be accounted for by the surround responses of ON and OFF PYs shown in Fig 4 as this would give the oppositely directed change in the RC firing rate for surround stimulation. Instead the observed response changes must mainly stem from center responses of laterally shifted PYs, i.e., with their receptive-field centers positioned in the surround of the RC cell. Note that in the present model example each RC receives feedback from a set of 2 × 2 PYs (each with elongated receptive-field centers as seen in panel A).

**Fig 13:**
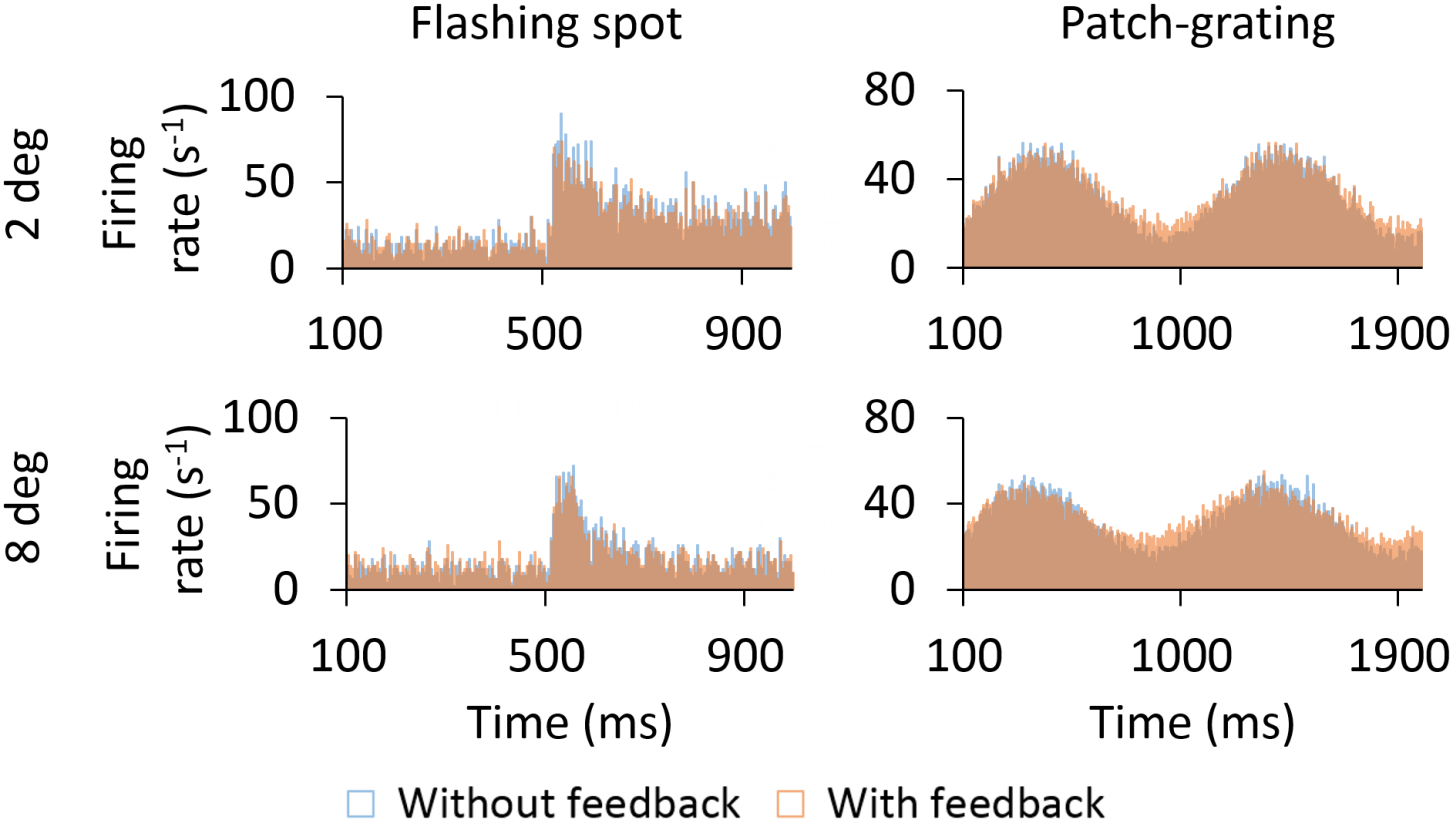
PSTHs of RCs for flashing spots and patch gratings with phase-reversed feedback and without feedback. Trial-averaged PSTHs of ON-center RC for two spot/patch diameters: 2 and 8 degrees. PSTHs for the phase-reversed feedback are compared with PSTHs shown in Fig 6. Corticothalamic synapse weights for the feedback configuration are the same as in Fig 10.

*Poststimulus time histograms (PSTHs):* We finally explored the effect of phase-reversed cortical feedback on PSTHs, both for flashing-spot and patch-grating responses. Fig 13 shows results for two spot/patch diameters: the smallest (2 degree diameter) essentially covering the receptive-field center, the largest (8 degree diameter) covering a large part of the surround. A detailed comparison of the cases with and without cortical feedback is difficult just by visual inspection. However, the key point is that the amplitude of the sinusoidal rate modulation is reduced with cortical feedback for large patch gratings (cf. Fig 10), which is clearly discernible. We also observe that the patch-grating response for the case with cortical feedback is phase-advanced compared to no-feedback case, in accordance with previous observations of the effect of inhibition-dominated feedback on the drifting-grating response transfer function [85].

#### Phase-matched (push-push) cortical feedback

**Fig 14:**
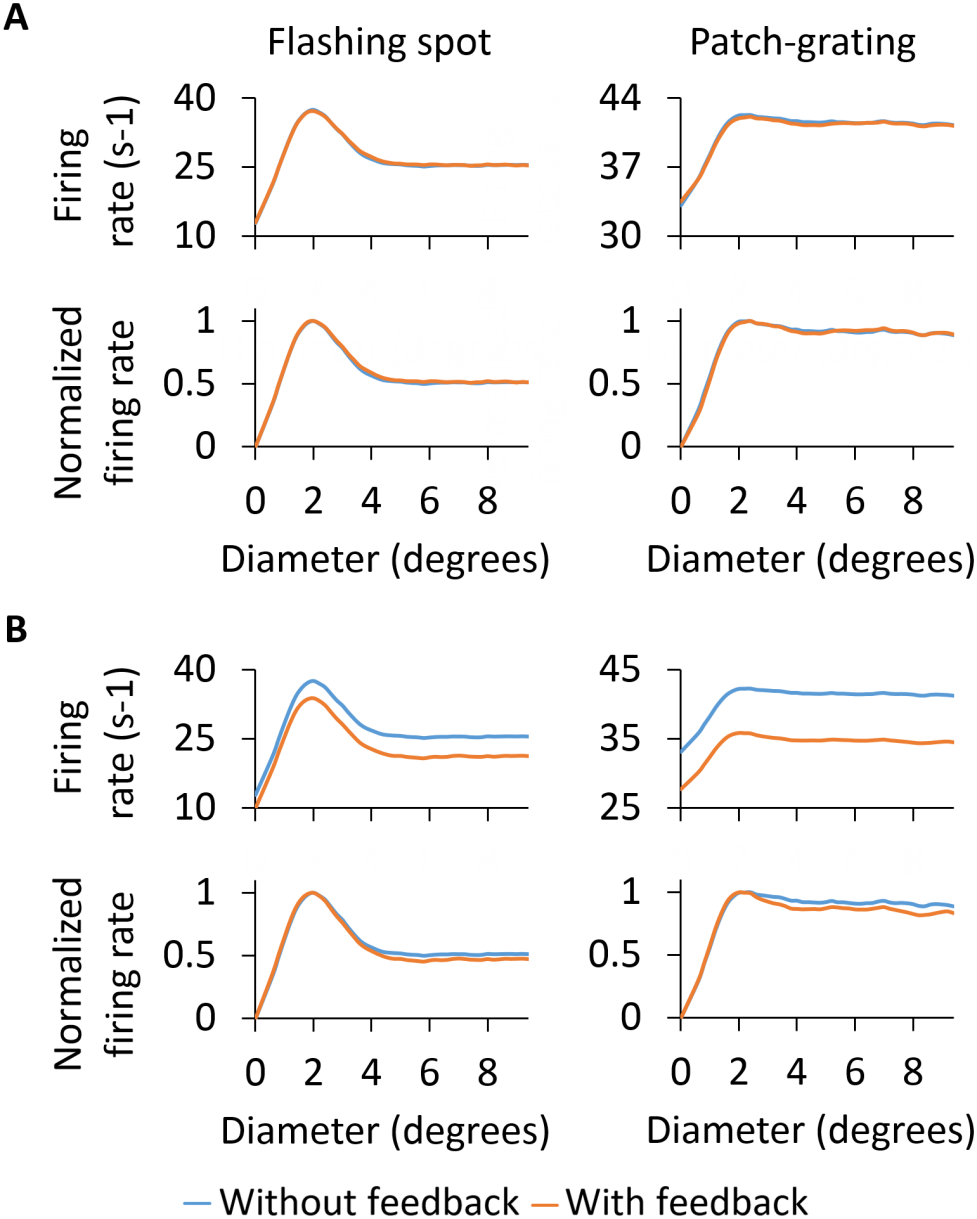
Area-response curves for ON-center RC with and without feedback for phase-matched and inhibitory-only feedback. A: phase-matched: synaptic weight between PYs and RCs set to 1.5 nS, between PYs and INs to 0.3 nS. B: inhibitory-only: synaptic weight between PYs and RCs set to zero, between PYs and INs to 0.3 nS The RC receives feedback from a region of 2 × 2 PYs.

*Area-response curves:* In the phase-matched configuration, both ON RCs and ON INs receive feedback from ON PYs (Fig 3A). Fig 14A shows the area-response results obtained for the same set of parameters used for the phase-reversed situation depicted in Fig 10. These parameter values roughly balance the excitatory and inhibitory feedback effects to RCs. Since these two effects have similar size but have opposite signs, the net effect on the RC for phase-matched feedback is as expected practically negligible both for flashing spots and patch gratings. As a consequence, the center-surround antagonism *α* is now essentially unaffected by the feedback.

We find that in order for cortical feedback to increase surround suppression in the RC response in this phase-matched configuration, the inhibitory contribution to the feedback must be larger than the excitatory contribution. To illustrate this point we show in Fig 14B area-response curves for the case when the excitatory feedback is turned off, i.e., the synaptic weight between PYs and RCs is set to zero. In this situation the cortical feedback again increases surround suppression on RCs, i.e., *α* is increased compared to the RC curve without feedback, from 50.1% to 54.6% for the flashing spot and from 11.2% to 18.3% for the patch grating (see Table 4). However, the surround suppression is still smaller than for the phase-reversed feedback (Fig 14) where the excitatory feedback from OFF PYs adds to center-surround suppression, not subtracts from it as for feedback from ON PYs in the phase-matched situation.

**Fig 15:**
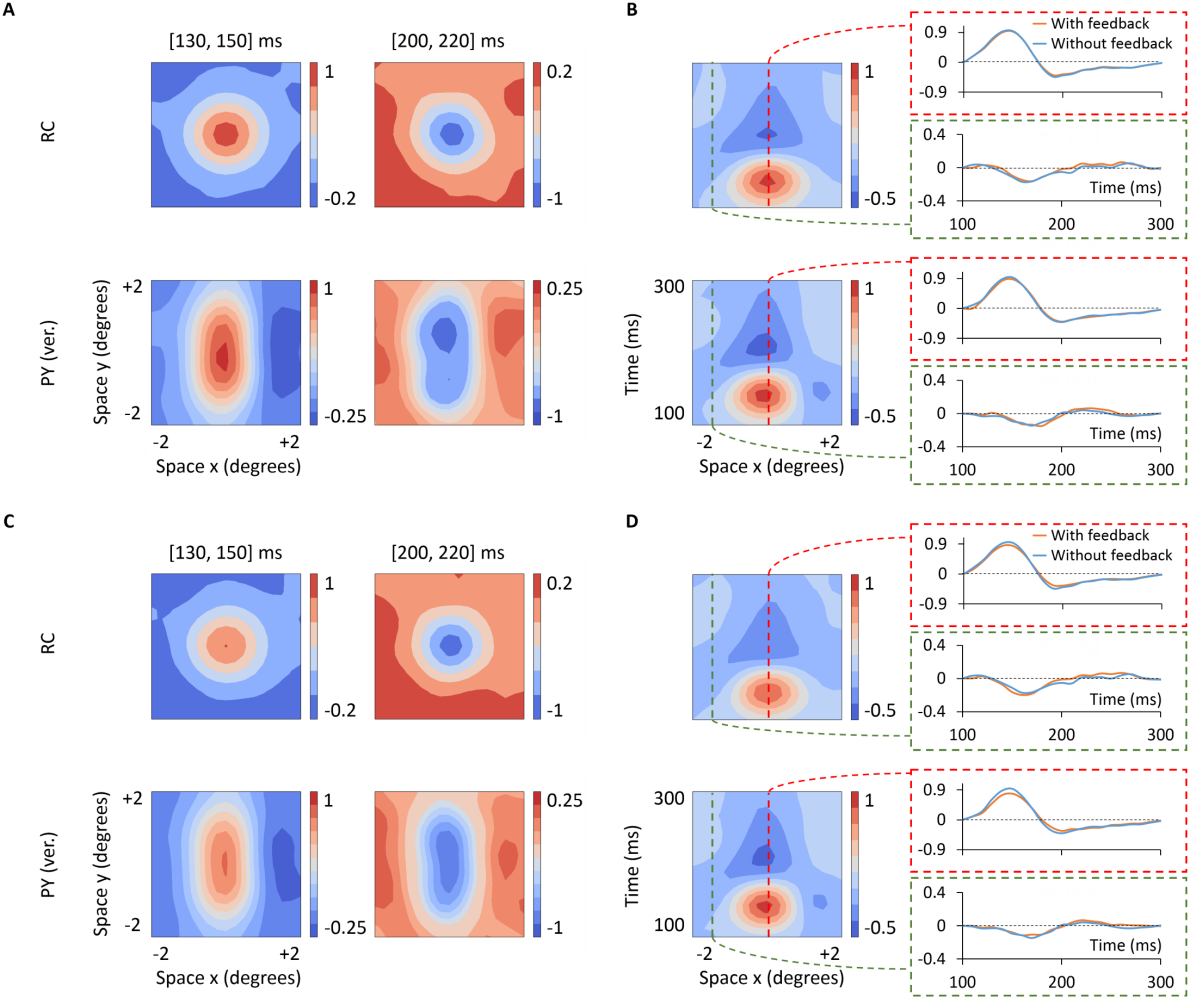
Spike receptive fields for RC and PY for phase-matched and inhibitory-only feedback. A–B: phase-matched: synaptic weight between PYs and RCs set to 1.5 nS, between PYs and INs to 0.3 nS. C–D: inhibitory-only: synaptic weight between PYs and RCs set to zero, between PYs and INs to 0.3 nS. The RC receives feedback from a region of 2 × 2 PYs. *x*-*y*-*t* receptive-field maps of ON-center RC and ON-center vertically oriented PY are shown in A and C. Their corresponding spatiotemporal *x*-*t* receptive-field profiles are shown in B and D. Additional column on the right of panels B and D shows 1D temporal profiles extracted from two locations of the *x*-*t* receptive field corresponding to the receptive-field center (ON subregion) and receptive-field surround (OFF subregion). Firing rates are normalized by the same values used in Fig 4.

*Spike receptive fields:* The spike receptive-field plots in Fig 15 further illustrate this point. For the case with phase-matched feedback effects of the cortical feedback are almost absent both for the center and the surround responses (panel B). With only inhibitory feedback present, the center response is reduced as for the phase-reversed situation in Fig 12. However, the extent of the reduction is smaller.

#### Influence of corticothalamic synapse weights and spatial connectivity profile of cortical feedback

**Fig 16:**
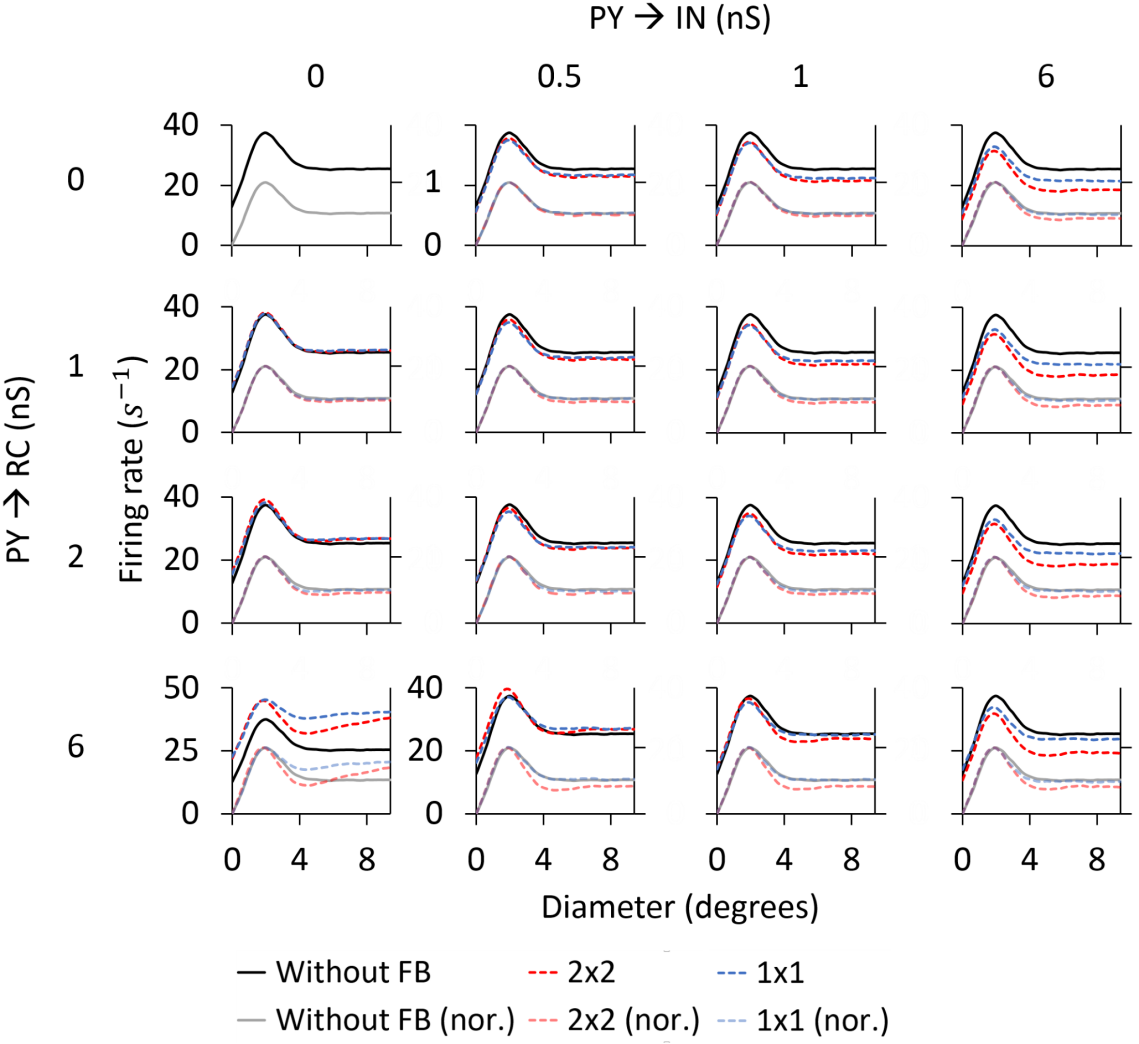
RC area-response curves for flashing spots for phase-reversed feedback. Normalized and unnormalized RC responses for different synapses weights between PYs and dLGN neurons, ranging from 0 (without feedback) to 6 nS, and for the two feedback spatial kernels: 1 × 1 and 2 × 2. Values shown for the synaptic weights represent the sum of all individual synaptic conductances of the same type converging to a given cell, i.e., for the 2 × 2 kernel, the value of every monosynaptic connection is the value depicted here divided by 4. The primary vertical axis of every panel (on the left) shows the values of the unnormalized response and the secondary vertical axis (on the right), the values of the normalized response, as shown for the panel in the first row and first column.

**Fig 17:**
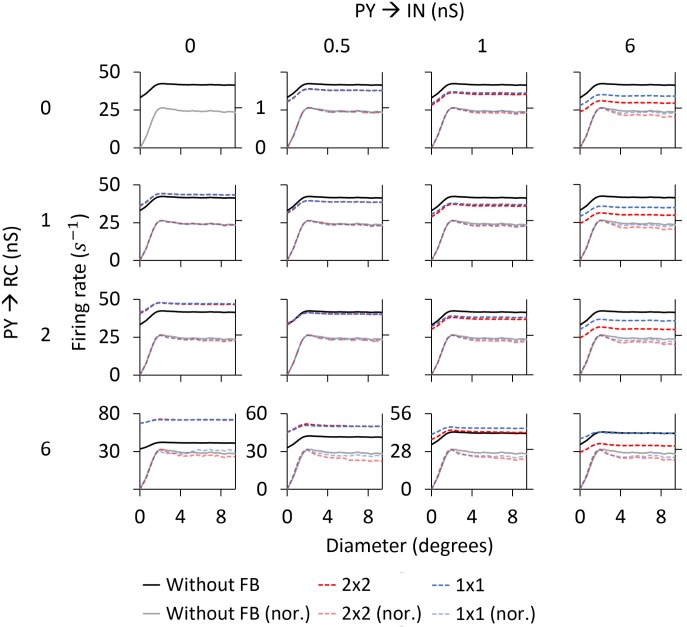
RC area-response curves for patch gratings for phase-reversed feedback. Normalized and unnormalized RC responses for different synapses weights between PYs and dLGN neurons, ranging from 0 (without feedback) to 6 nS, and for the two feedback spatial kernels: 1 × 1 and 2 × 2. Values shown for the synaptic weights represent the sum of all individual synaptic conductances of the same type converging to a given cell, i.e., for the 2 × 2 kernel, the value of every monosynaptic connection is the value depicted here divided by 4. The primary vertical axis of every panel (on the left) shows the values of the unnormalized response and the secondary vertical axis (on the right), the values of the normalized response, as shown for the panel in the first row and first column.

**Fig 18:**
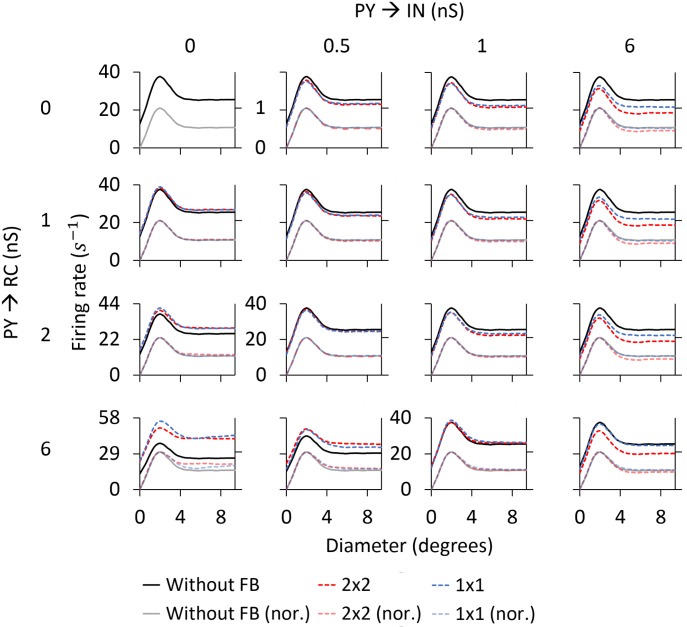
RC area-response curves for flashing spots for phase-matched feedback. Normalized and unnormalized RC responses for different synapses weights between PYs and dLGN neurons, ranging from 0 (without feedback) to 6 nS, and for the two feedback spatial kernels: 1 × 1 and 2 × 2. Values shown for the synaptic weights represent the sum of all individual synaptic conductances of the same type converging to a given cell, i.e., for the 2 × 2 kernel, the value of every monosynaptic connection is the value depicted here divided by 4. The primary vertical axis of every panel (on the left) shows the values of the unnormalized response and the secondary vertical axis (on the right), the values of the normalized response, as shown for the panel in the first row and first column.

**Fig 19:**
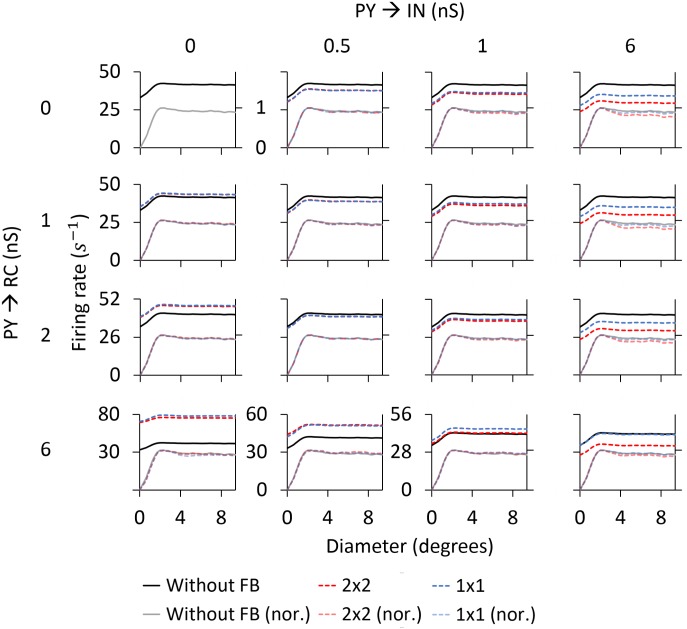
RC area-response curves for patch-grating for phase-matched feedback. Normalized and unnormalized RC responses for different synapses weights between PYs and dLGN neurons, ranging from 0 (without feedback) to 6 nS, and for the two feedback spatial kernels: 1 × 1 and 2 × 2. Values shown for the synaptic weights represent the sum of all individual synaptic conductances of the same type converging to a given cell, i.e., for the 2 × 2 kernel, the value of every monosynaptic connection is the value depicted here divided by 4. The primary vertical axis of every panel (on the left) shows the values of the unnormalized response and the secondary vertical axis (on the right), the values of the normalized response, as shown for the panel in the first row and first column.

In the following, we further investigate the behavior of the network model by exploring the dependence of the area-response curves on the model parameters describing the cortical feedback. We systematically varied weights of synapses between cortical PYs and RCs and INs. Simulations were done for both feedback arborization configurations, 1 × 1 and 2 × 2, and also for the different phase arrangements between receptive fields of PYs and dLGN neurons, phase-reversed (Figs 16 and 17) and phase-matched (Figs 18 and 19).

In Fig 16, we show the different RC responses to the flashing spot for the phase-reversed case. As expected the overall firing rates of RCs are increased when increasing the feedback weight values for RCs (moving down) and are reduced when increasing the values for INs (moving right). This is seen for both spatial kernels, 1 × 1 and 2 × 2. However, the 2 × 2 feedback configuration is seen to increase surround suppression more than 1 × 1.

The upper row of panels corresponds to the case where there is no feedback excitation from PYs to RCs and clearly illustrates how inhibitory feedback increases the surround suppression of RCs. The first column of panels instead shows the case where feedback inhibition is turned off, and there is only feedback excitation. Here it is seen that very large values of the excitatory connection from PYs to RCs (cf. row 4) can even result in an opposite effect, i.e., a reduced surround suppression. However, the combined effect of feedback excitation and inhibition enables a larger increase of the surround suppression compared to the case with only feedback inhibition as exemplified by the lower right panel (row 4, column 4) in Fig 16.

In general terms, a similar behavior is observed for the responses to the patch grating with phase-reversed feedback (Fig 17): there are larger firing rates when increasing the excitatory connection and a marked reduction of firing rates for the greatest values of the IN synaptic conductance. Also here the combined effect of excitation and inhibition from cortical feedback produces the largest increase in surround suppression of RCs.

RC responses with the phase-matched configuration are shown for the flashing spot in Fig 18 and for the patch grating in Fig 19. Here the area-response curves with feedback largely maintain the same shape of the area-response curves without feedback since both cortical excitation and inhibition are driven by the same type of cell. Unlike for the phase-reversed feedback, the level of surround suppression in the RC is only increased when the excitatory feedback is absent, i.e., top rows in Figs 18 and 19. With excitatory feedback added (rows 2–4), the surround suppression is reduced. Thus with excitatory feedback present in addition to feedback inhibition, the surround suppression is always smaller for the phase-matched set-up compared to the phase-reversed set-up.

**Fig 20:**
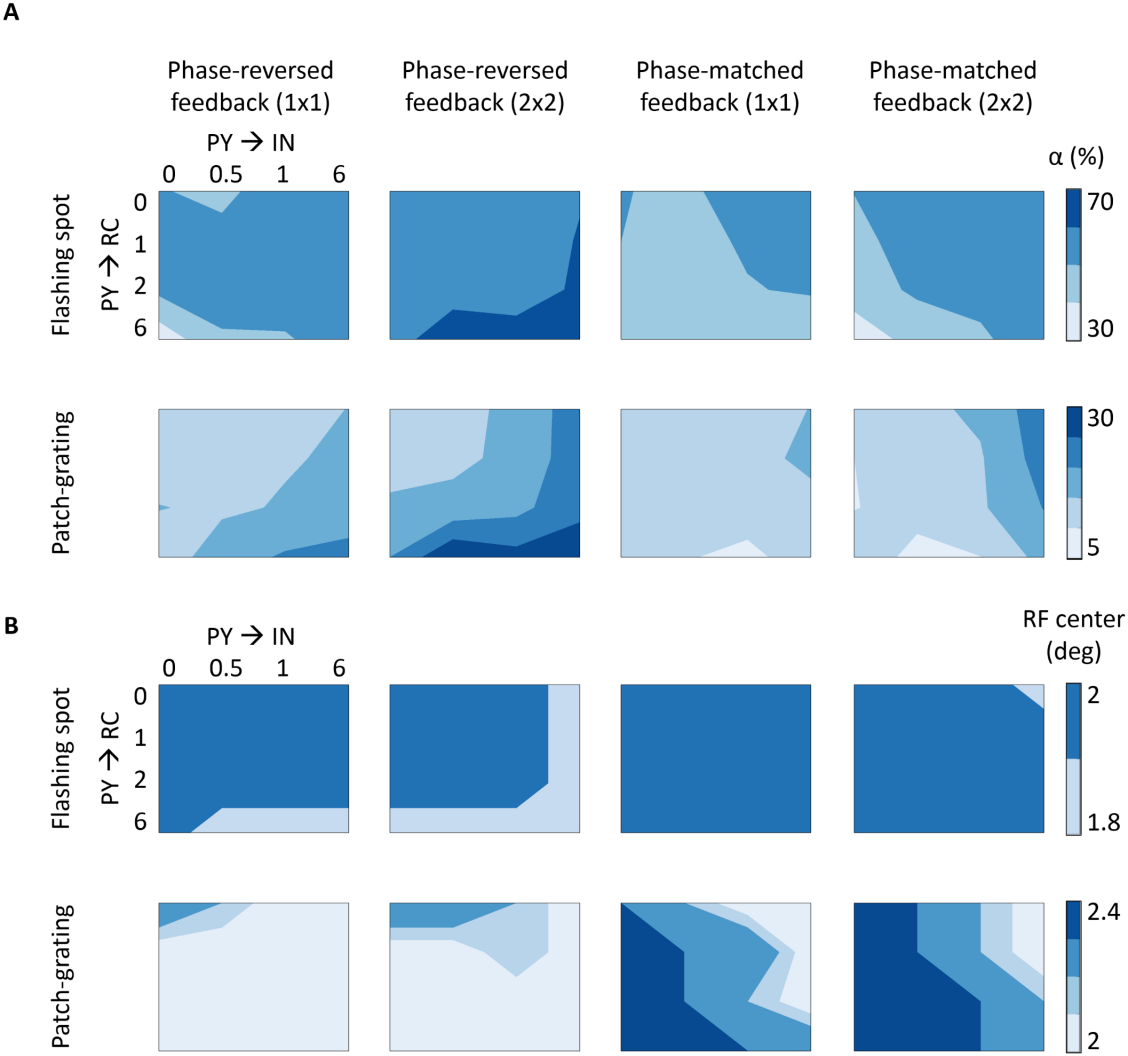
Summary of response measures for area-response curves. Contour plots of the center-surround antagonism coefficient (A) and stimulus diameters giving the largest response (corresponding to the receptive-field (RF) center for flashing spot) (B) as a function of synaptic weights and feedback spatial extent for the two phase arrangements considered: phase-reversed and phase-matched.

Two measures have been commonly been used to characterize area-response curves: the diameter giving the largest response (corresponding to the receptive-field center size for flashing spots) and the center-surround antagonism [27, 28, 41]. In Fig 20 we show a summary of these response measures from the previous area-response curves (Figs 16–19).

We first consider the effects of cortical feedback on the center-surround antagonism coefficient (Fig 20A). Independently of the type of stimulus, there is a significant difference between the phase-reversed and phase-matched feedback configurations: in the phase-reversed case, high values of the center-surround antagonism coefficient are achieved by those parameter combinations that exert both strong excitation and inhibition to the RC (towards the bottom right corner), whereas, in the phase-matched case, only large values of inhibition can increase the center-surround antagonism coefficient (towards the top right corner).

For the same synaptic conductances for the feedback, the phase-reversed arrangement always gives the largest values of the center-surround antagonism coefficient. Further, with this configuration, cortical feedback always increases the center-surround antagonism more for the patch-grating than for the flashing spot. We also see that the wider feedback axonal arborization, i.e., 2 × 2, always gives larger surround suppression than the narrow 1 × 1.

The sizes of the spot/patch that produce the maximal RC response are shown in Fig 20B. For the phase-reversed feedback, a reduction of the maximum-response size is seen for the patch grating when one or both types of cortical feedback is present. The same tendency, though less prominent, is seen also for flashing spots. Also for the phase-matched feedback, the maximum-response sizes are reduced by increasing inhibition to RCs. However, in the phase-matched case, excitatory feedback had the opposite effect compared to the phase-reversed case, i.e., excitation enlarged the maximum-response sizes.

## Discussion

In the present paper we have developed a mechanistic network model of the thalamocortical system with explicit representations of LGN cells (relay cells (RCs) and interneurons (INs)) and orientation-selective layer 6 simple cells placed on two-dimensional spatial grids. The LGN and cortical cells are represented by biophysical neuron models based on the cable equation and Hodgkin-Huxley type active conductances. The input of the model is provided by retinal ganglion cells (GCs) implemented by means of descriptive filter models.

The main focus of the study has been exploration of the effects of cortical feedback on the spatial responses of RCs to flashing-spot and patch-grating stimuli as this has received substantial experimental attention [2, 4, 19, 32]. Comparison of our simulation results with previous experimental findings supports the notion that a ‘push-pull’ (phase-reversed) organization of cortical feedback [62], i.e., ON-center RCs receive direct (monosynaptic) excitatory feedback from OFF-dominated cortical cells and indirect inhibitory feedback from ON-dominated cortical cells, provides a dual effect that simultaneously amplifies excitatory responses in the receptive-field center and inhibitory responses in the receptive-field surround of RCs [18, 83]. As a result, the center-surround antagonism of RCs is amplified by cortical feedback and the maximum RC response occurs for reduced stimulus sizes. The combination of these two effects, excitatory in the receptive-field center [18] and inhibitory in the receptive-field surround [4, 19], may be understood as complementary functions that dynamically sharpens the spatial focus of the receptive field and increase their spatial resolution.

### Model construction and validation

#### Feedforward model

The present work builds on our previous feedforward model that investigated the roles of triadic and axonal inhibition from dLGN INs on the RC response [41]. In the previous model, a single multicompartmental IN model incorporating dendrodendritic interaction between RCs and INs on triads [45] was used in combination with five single-compartment RC point-neuron models (adapted from [42]). Further, the parameters of the synaptic connections were fitted so that the model predicted flashing-spot area-response curves for RCs and GCs in accordance with experimental data from cat dLGN [27, 28]. In the present model the connectivity pattern for retinogeniculate and intrageniculate connections in [41] is kept. The plausibility of the RC and GC models was supported by the observation that their spatiotemporal receptive-field profiles (upper two rows of panels in Fig 4) were seen to be qualitatively similar to experimental observations [26].

With the present focus on how cortical feedback affects the RC response, we constructed a minimal model of layer 6 in the primary visual cortex including a single type of cortical cells, pyramidal cells (PYs). Further, the model in [41] was extended to include both ON- and OFF-center cells to allow for cross-symmetry thalamocortical and corticothalamic projections. Receptive fields of simple cortical cells are orientation-selective, and two orientation-selective cortical populations have been included in the model, one preferring horizontally-oriented stimuli, the other vertically-oriented stimuli. This orientation selectivity was constructed by tailoring thalamocortical excitatory inputs from 3 ON and 3 OFF RCs, each ON and OFF subregion spanning a patch of 3 deg × 1 deg in the visual field with a length/width ratio of about 2.5 [58–61] (Fig 1).

The resulting PY spatiotemporal receptive-field profiles was observed to resemble the experimentally-observed receptive field for the ‘separable simple cell’ in [26] (Fig 4). We further computed two receptive-field measures as described in [48, 69]: an overlap index (Eq 13) assessing the spatial segregation of subregions within the receptive field and a push-pull index (Eq 14) determining the relative weight of the antagonistic response to stimuli of opposite contrast, and confirmed that they were compatible with what has been observed for cortical simple cells [48, 69].

#### Feedback model

The detailed arrangement of the corticothalamic feedback provided by layer-6 cells is less known. We thus investigated several candidate feedback configurations both in terms of (i) the different phase arrangements from the ON and OFF zones in the visual cortex to the dLGN cells and (ii) the spatial divergence of the feedback. With regard to the phase arrangements between receptive fields of cortical cells and LGN cells, we have considered two different patterns (Fig 3): In the phase-reversed arrangement (‘push-pull’) [62], ON-center PYs synapse on ON-center INs while OFF-center PYs synapse on ON-center RCs. In the phase-matched arrangement (‘push-push’), both ON-center INs and ON-center RCs receive feedback from ON-center PYs.

Previous studies (on cat LGN) have indicated that most interneuron action potentials can be accounted for by retinal input [64, 65]. Therefore, we chose to put cortical synapses distally on INs. With this setup, cortical feedback could increase the inhibition of RCs via dendrodendritic interaction with little effect on the IN firing rate (cf. Fig 11).

In terms of the spatial divergence of the corticothalamic axons, we have analyzed two feedback configurations: 1 × 1 and 2 × 2. In the 1 × 1 feedback, every PY synapses a single spatially overlapping RC and the corresponding IN dendrite. In the 2 × 2 feedback, every PY connects to four neighboring RCs and the four dendrites of a single IN. Such a spatially extended arrangement (2 × 2) is more in accordance with anatomical observations of the spatial spread of corticothalamic axons in cat dLGN [63].

### Area-response curves

The main results from our model study were the area-response curves for flashing-spot and patch-grating stimuli, a commonly used measure of visual responses for cells in the early stages of the visual system [2, 4, 18, 19, 27, 28, 40, 80, 81].

We first considered the case with a rough balance between excitatory and inhibitory feedback so that the main effect of cortical feedback is on the shape of the area-response curves, not the magnitude (Figs 10 and 14). With a phase-reversed feedback arrangement a clear feedback-induced increase in surround suppression is observed both for flashing spots and patch gratings (Fig 10), as quantified by the center-surround antagonism coefficient *α* (Eq 12) (Table 4). Such a feedback-induced increase of surround suppression has been observed in experiments with both flashing spots [32] and patch gratings [4, 19], although the effect appears more significant for patch gratings [2, 4]. Our model results gave a larger increase of surround suppression for the patch-grating stimulus, but not as prominent as the increase reported by Sillito et al. [4]. With the same choice of parameters, a phase-matched feedback arrangement resulted in very little change in surround suppression for both types of stimulus (Fig 14).

Increased surround suppression implies that RC cells in relative terms become more responsive to small stimuli and, thus, the cell more selective in spatial tuning. An additional effect of the phase-reversed feedback is the shrinking of the stimulus size giving the maximum responses in the area-response curves, clearly observed for the phase-reversed feedback, but largely absent for phase-matched feedback (Figs 10 and 14).

We next did a parameter sweep, i.e., investigated the effects of cortical feedback on the RC area-response curves for a wide range of different synaptic weights between PYs and dLGN neurons and for the different spatial feedback kernels (1 × 1 and 2 × 2) (Figs 16–19). The results for our two key area-response curve measures, the stimulus diameters giving the largest response and the center-surround antagonism coefficient *α*, were summarized in Fig 20.

A first observation was that both for flashing-spot and patch-grating stimuli, the phase-reversed and phase-matched cases gave very different dependency of the center-surround suppression, i.e., center-surround antagonism coefficient *α*, on synaptic weights (Fig 20A). For the phase-reversed case, high values of the center-surround antagonism coefficient were achieved by those parameter combinations that exert both strong excitation and (indirect) inhibition to the RC (towards the bottom right corner). Here the ON-center inhibition and the OFF-center excitation both contribute to increasing the surround suppression. Thus large values of the surround suppression can be achieved even when excitatory and inhibitory effects are roughly balanced [18, 83]. In contrast, for the phase-matched case, feedback-induced increases in the center-surround antagonism coefficient *α* required the inhibition to dominate the excitation. This reflects that the effects of ON-center inhibition and ON-center excitation in the feedback tend to cancel each other out. This is in accordance with the observation in Figs 10 and 14 where the area-response curve for the ‘inhibition-only’ case was seen to represent an intermediate case between the phase-reversed and phase-matched situations.

When comparing the different spatial feedback patterns for the phase-reversed case, the 2 × 2 feedback pattern was seen to be more effective in increasing surround suppression in the RC response than the 1 × 1. Incidentally, a spatially widespread feedback pattern has been suggested by anatomical studies of the innervation pattern of corticothalamic axons in the dLGN [63].

For flashing-spot stimuli only small variations in the diameters producing the maximal RC response were observed when varying the synaptic weights (Fig 20B). However, for patch-grating stimuli a reduction was observed in the maximum-response diameter was observed when one or both types of cortical feedback were present.

### Comparison with previous modeling approaches

Other modeling studies have also investigated the effect of cortical feedback on spatial processing of RCs with different stimulus patterns [39, 40]. The focus in [39] was on exploring the role of cortical feedback in modulating RC responses to discontinuity in orientations in gratings in bipartite stimuli. In [40] the extended DOG (eDOG) model was introduced, allowing for analytical explorations of effects of cortical feedback in certain settings, i.e., with certain combinations of excitatory and (indirect) inhibitory feedback from ON- and OFF-center cortical cells onto RCs. There a preliminary use-case showed that a phase-reversed (‘push-pull’) arrangement of cortical feedback where ON-center RCs receive direct excitation from OFF-driven cortical cells and balanced indirect inhibitory feedback from ON-driven cortical cells, may provide increased center-surround antagonism.

Our biophysical model and the above-discussed firing-rate models represent opposite extremes in terms of biological detail in LGN circuit models [86]. Models at an intermediate complexity level where the cells are modeled as integrate-and-fire neurons have also been used to explore cortical feedback effects on LGN cell [33–36]. However, these have focused on temporal response properties such as feedback-induced spike synchronization [35], long-lasting correlations [36] and effects of feedback on visual latency [33], not the spatial properties which has been the main topic here.

### Future model applications and model extensions

An obvious next application of the present model would be to explore temporal response properties of LGN cells and, in particular, how these are affected by various types of cortical feedback. One line of inquiry would be to explore the relative roles of feedforward and feedback connections in shaping the temporal receptive fields of LGN cells, analogous to the questions addressed by the firing-rate models in [37] and [38]. Another line of research would be on studying spike synchronicity and correlations as addressed earlier with integrate-and-fire models [35, 36]. A third line could be to explore in detail how the temporal structure of the PSTH, and in particular the ‘interval histogram’ of RC spikes, is affected by feedback [34].

In addition to feedback from cortex, both RCs and INs receive inhibitory feedback from neurons in the thalamic reticular nucleus (TRN) [5]. TRN neurons are thought to play a key role in the process of sleep spindle oscillations generated within the thalamic circuitry [42, 43]. The TRN also contributes to the control of visual attention and awareness [87], but the effects on procession of visual signals remain poorly understood [88]. TRN neurons do not receive direct input from the retina as LGN INs, instead they receive feedforward visual signals from collaterals of geniculocortical axons. TRN neurons also receive cortical feedback through corticothalamic axons, and their synapses on RCs are situated in close proximity to those of corticothalamic axons [1]. Given this organization of synaptic connections and its position within the network, TRN cells are likely to influence the transfer of visual information in a different manner than LGN INs. Modeling studies exploring the putative role of TRN neurons on visual processing have already been pursued [89], and the present biophysical model could be extended to include also such neurons when more is known about these neurons and their possible role in visual processing.

The present model assumes static synapses while a number of studies have demonstrated short-term plasticity in different synapses of the thalamocortical circuit, i.e., short-term depression at the retinogeniculate [90, 91] and geniculocortical [92, 93] synapses, as well as in the feedback connection from cortex to INs [94]. In contrast, the feedback connection from cortex to RCs appears to be facilitating [90, 95]. Such plasticity opens up for an even richer dynamical repertoire of the circuit, and would be an interesting topic for a future study using the present model with static synapses as a starting point. In particular, it would be interesting to explore if short-term synaptic plasticity could affect our prediction that phase-reversed cortical feedback is the most effective mechanism for increasing center-surround antagonism.

dLGN cells have two different response modes, burst and tonic, suggested to relate of the animal [5, 96, 97]. Modulatory inputs from other parts of the brain may switch between these modes by shifting the baseline membrane potentials of RCs and INs. Tonic firing has been suggested to be more suitable for transferring visual information because it avoids nonlinear distortions created during burst firing, while burst firing was suggested to be best suited as an ‘alarm clock’, i.e., rapid stimulus detection [5]. Recent studies have demonstrated, however, that thalamic bursts can also contribute to sensory processing [98–101]. In the current study, our RC and IN models were based on data from dLGN neurons that rested on relatively depolarized membrane potentials, -60 mV and -63 mV, respectively, and fired predominantly in the tonic mode (Fig 2). An exploration of the functional roles of the two firing modes, and putative switches between them, would be another natural extension of the present work.

The present model of primary visual cortex is obviously simplified. Cells in layer 4 of cortex are the main targets of projections from RCs, while the feedback from cortex to dLGN comes from cells in layer 6. Even though there are also projections from RCs to layer-6 cells, there are likely cross-layer processing in cortex that affects the thalamocortical feedback loop and difficult to capture by a single-layer cortex model. Despite the model simplicity, the pyramidal-cell receptive fields produced by our network model (Figs 4, 12 and 15) are nevertheless seen to resemble the receptive fields of simple cells which also has been observed in layer 6 of cat visual cortex [102]. Thus the error introduced by our simplified cortical network model could be modest for the present application, but this needs further exploration when thalamocortical models including more comprehensive cortical circuitry becomes available.

Further, there are several neural mechanisms that our simplified model of cortical orientation tuning does not account for, such as recurrent cortical excitation or horizontal inhibitory connections [58, 103–105], which can amplify a weak orientation bias. Although the area-response curves of cortical cells to the patch grating in Figs 8 and 9 showed a marked difference for gratings at preferred and non-preferred orientations, stimuli presented at non-preferred orientations did not suppress cortical response to the background rate as observed experimentally in some cells [106]. A stronger orientation selectivity of the cortical cells would likely affect the feedback-induced changes in RC response, but how, and to what extent, remains to be explored.

While one option for extending the present model would be to add more neuron types to a single-layer cortex model, it might be tempting to aim to connect the present biophysically detailed model for the dLGN circuit with an equally detailed model for the primary visual cortex. However, at present such models are lacking, and a comprehensive model based on biophysical neuron models including both the dLGN and, say, V1 would anyway be computationally extremely demanding. An alternative could be to instead model V1 dynamics with simpler neuron models such as the Potjans-Diesmann network model based on integrate-and-fire neurons [107].

Experimental studies of cortical feedback effects on response properties in the dLGN have been ongoing for at least 40 years (see, e.g., [7]). However, a recurring challenge has been to reversibly remove cortical feedback in a controlled manner to compare physiological responses of dLGN cells with and without cortical feedback. Both cooling [11] and pharmacological manipulations [18] have been used. However, the advent of optogenetics now offers unprecedented opportunities for highly-controlled activation or deactivation of individual cell types. In [108] the role of layer-6 cells in providing gain control for the visual responses in the upper layers of mouse visual cortex was studied by such techniques. A similar study where visual responses of dLGN cells are measured while the corticothalamic cells in layer 6 are selectively activated or deactivated by photostimulation, would be most welcome for testing predictions of the present model.
